# Dielectric screening in perovskite photovoltaics

**DOI:** 10.1038/s41467-021-22783-z

**Published:** 2021-04-30

**Authors:** Rui Su, Zhaojian Xu, Jiang Wu, Deying Luo, Qin Hu, Wenqiang Yang, Xiaoyu Yang, Ruopeng Zhang, Hongyu Yu, Thomas P. Russell, Qihuang Gong, Wei Zhang, Rui Zhu

**Affiliations:** 1 State Key Laboratory for Artificial Microstructure and Mesoscopic Physics, School of Physics, Frontiers Science Center for Nano-optoelectronics & Collaborative Innovation Center of Quantum Matter, Peking University, Beijing, China; 2 Department of Electrical Engineering, Princeton University, Princeton, NJ USA; 3 School of Microelectronics, Southern University of Science and Technology, Shenzhen, China; 4 Polymer Science and Engineering Department, University of Massachusetts, Amherst, MA USA; 5 Materials Sciences Division, Lawrence Berkeley National Laboratory, Berkeley, CA USA; 6 National Center for Electron Microscopy, Molecular Foundry, Lawrence Berkeley National Laboratory, Berkeley, CA USA; 7 Collaborative Innovation Center of Extreme Optics, Shanxi University, Taiyuan, China; 8 Peking University Yangtze Delta Institute of Optoelectronics, Nantong, Jiangsu China; 9 Advanced Technology Institute, University of Surrey, Guildford, UK; 10 State Centre for International Cooperation on Designer Low-Carbon and Environmental Material (SCICDLCEM), School of Materials Science and Engineering, Zhengzhou University, Zhengzhou, China

**Keywords:** Solar cells, Solar energy and photovoltaic technology

## Abstract

The performance of perovskite photovoltaics is fundamentally impeded by the presence of undesirable defects that contribute to non-radiative losses within the devices. Although mitigating these losses has been extensively reported by numerous passivation strategies, a detailed understanding of loss origins within the devices remains elusive. Here, we demonstrate that the defect capturing probability estimated by the capture cross-section is decreased by varying the dielectric response, producing the dielectric screening effect in the perovskite. The resulting perovskites also show reduced surface recombination and a weaker electron-phonon coupling. All of these boost the power conversion efficiency to 22.3% for an inverted perovskite photovoltaic device with a high open-circuit voltage of 1.25 V and a low voltage deficit of 0.37 V (a bandgap ~1.62 eV). Our results provide not only an in-depth understanding of the carrier capture processes in perovskites, but also a promising pathway for realizing highly efficient devices via dielectric regulation.

## Abstract (web summary)

Performance of perovskite photovoltaics is greatly affected by undesirable defects that contribute to non-radiative losses. Here, the authors mitigate these losses by doping perovskite with KI to alter the dielectric response, thus defect capturing probability, resulting in inverted device with PCE of 22.3% and low voltage loss.

## Introduction

Over the last decade, perovskite photovoltaics (PPV) have shown dramatic achievements in both the device efficiency and long-term stability^1–7^. Despite notable advances in the device performance, there is still a certain gap between the record power conversion efficiencies (PCEs) and the theoretical limit defined by the Shockley-Queisser theory. The key factor is ascribed to the nonradiative recombination losses related to defects within the bulk of perovskite films and at the contact interfaces^8–11^. To further push PCEs toward the Shockley-Queisser limit, it is imperative to understand defects and the mechanism of the electron or hole capture by the defects^12^ (a predominant contribution to nonradiative recombination losses) so as to develop effective strategies for suppressing these undesirable loss pathways. In general, the presence of defects in the lattice can increase the scattering event of charge carriers during the transport process, thereby influencing the phonon energies and the strength of electron–phonon coupling^13^. Under the conditions of light soaking and/or external bias, defects have proven to be the major causes of the ion migration within the perovskite films and poor device stability^14,15^. Hence, eliminating the adverse effects induced by defects is of paramount importance to maintain high device efficiencies and long-term operational stabilities of the PPV.

Defect passivation strategies and their positive effects on device performance have been extensively studied in the PPV community^16–18^. Nevertheless, an in-depth understanding of charge carriers (including both electrons and holes) and defects, along with electron–phonon coupling has been rarely discussed. In traditional semiconductors, photoexcited electrons or holes generally undergo Coulomb interactions with positively and/or negatively charged defects surrounding them^19,20^. Coulomb attraction or repulsion depends on the types of defects they meet during the transport process. It is expected that Coulomb attraction between the defects and carriers increases the defect capture cross-section (representing the probability of carriers being captured by the recombination center), while Coulomb repulsion decreases the capture cross-section^12^. In terms of intrinsic perovskite materials, Zhu et al. proposed that the carriers in metal halide perovskites can be protected from the scattering as large polarons^21^. Zhu et al. demonstrated the liquid-like molecular reorientation motions in perovskite crystals enable the efficient screening of charge carriers^22^. Anusca et al. explained that the screening induced defect tolerance stems from the reorientation of the organic dipoles and polaronic relaxation of ionic lattice^23^. Together, this means that the Coulomb interaction between carriers and defects can be managed by the screening effect, suppressing the adverse impact of defects in the intrinsic perovskites. However, to date, tuning the screening effect by material design with extrinsic compositional engineering and linking these changes with device performance have not been discussed.

Here, we report a dielectric-screening effect enabled by controlling the space charge within formamidinium-cesium lead halide perovskites, and for the first time, we provide a coherent picture coupling the screening effect and device performance. Beginning with the localized Coulomb interactions, we theoretically demonstrate that controlling dielectric response is a feasible pathway to achieve screening with consideration of defect capture cross-section. Through the incorporation of potassium-based species into the bication perovskites, we find that the controlled dielectric responses of the perovskites can be effectively achieved. Changes in the dielectric responses of the perovskite films lead to a substantial variation of Coulomb interactions and defect capture cross-sections, thereby producing a dielectric-screening effect. Such an effect lowers the possibility that carriers are trapped in defects, thus, even if the defects still exist, they are, to some extent, “invisible” to charge carriers as if they are “stealth”. Further temperature-dependent photoluminescence measurements show an anomalously weaker electron–phonon coupling for the optimized perovskites. Based on these improvements, nonradiative recombination pathways can be considerably suppressed. As a result, we achieve high-performance inverted PPV devices with PCEs exceeding 22%, and a record open-circuit voltage (*V*_oc_, 1.25 V) for a bandgap of 1.62 eV. Furthermore, we quantify the specific voltage losses from the film to a full device, providing insights into the predominant voltage loss pathways in state-of-the-art PPV.

## Results

### The scheme of the dielectric-screening effect

Defects play vital roles in nonradiative recombination losses of semiconductors. With respect to defect-assisted recombination losses, a key process is electrons or holes captured by the nearest defects, and the probability of this process could be described by the defect capture cross-section. Taking the typical electron capture process as an example, when electrons meet positively charged defects, they can be captured by these positive defects by Coulomb attraction. In the case of Coulomb attractive defects, given that the required Coulomb potential energy equals to the thermal energy, we can get the capture cross-section for electrons *σ*_−_ as ref. ^12^:

1 $${\sigma }_{-}=\frac{{q}^{4}}{16\pi {({\varepsilon }_{\text{r}}{\varepsilon }_{0}{k}_{\text{B}}T)}^{2}},$$

where *q* is the elementary charge, *ε*_r_ and *ε*_0_ are dielectric constant and vacuum permittivity, respectively, *k*_B_ is the Boltzmann’s constant and *T* is the temperature. From the Eq. (1), we can see that, at a constant temperature, *σ*_−_ decreases as the dielectric constant increases, implying that an increase in the dielectric constant of the perovskite can result in the reduction of the defect capture cross-section. This change, as a kind of dielectric-screening effect, will weaken the trapping behavior of charge carriers by the defects and facilitate the favorable carrier transport. Note that here we only discuss electrons for simplicity, but the same principle applies to holes.

The dielectric constant is the ratio of the permittivity of a substance to the permittivity of vacuum. It describes the degree to which materials can contain an electric flux and is also frequency dependent. Generally, there are multiple dielectric response modes over a wide range of frequencies, namely, optical dielectric response (~10^14^ Hz) from electron density^24^, ionic dielectric response (~10^12^ Hz) from lattice vibration^25^, dipolar dielectric response (~10^9^ Hz) from dipolar species^26^, and space-charge dielectric response (<10^6^ Hz) from both electronic and/or ionic space charge^27^. The frequency dispersion relation of the complex dielectric constant ε^*^ for a certain response mode is given by the well-known Cole-Cole equation^28^:

2 $${\varepsilon }^{\ast }(\omega )={\varepsilon }_{\infty }+\frac{{\varepsilon }_{\text{s}}-{\varepsilon }_{\infty }}{1+{(\text{i}\omega {\tau }_{\text{c}})}^{1-\alpha }},$$

where *ε*_∞_ is the “infinite frequency” dielectric constant limit, *ε*_s_ is the static dielectric constant,*ω* is the angular frequency, *τ*_c_ is the relaxation time constant, and *α* is the parameter reflecting the width of relaxation time distribution^29^. Further details about this equation can be seen in Supplementary Note 1. The dielectric constant of a material can be tuned by varying the composition. Alkali halide species, in particular small alkali cations with large halide anions, are well-known to be highly polarizable with strong bonding strength^30^, and are already used to regulate dielectric properties in many different kinds of materials^31–33^. When adding alkali halide species to the metal halide perovskites, previous work has shown that small alkali cations and halide anions tend to interact at the grain boundaries while the latter fill the halide vacancies^34^. Such charged alkali cations with halide anions at the grain boundaries can cause the variation in the space charge, thus influencing the dielectric response at the space-charge region (below 10^6^ Hz as mentioned above) and ultimately varying the localized electric fields arising from charged defects^35^. Changes in the localized electric fields by the variation in the dielectric constant will lead to defect screening, which can mitigate the adverse effects of pristine unoccupied defects in the films. Thus, by rational selection of extrinsic addition with alkali halide species, effective screening of the charged defects at the grain boundaries can be achieved, leading to reduced defect capture cross-sections and improved charge-carrier transport.

### Controlled dielectric response for the perovskite

**Fig. 1:**
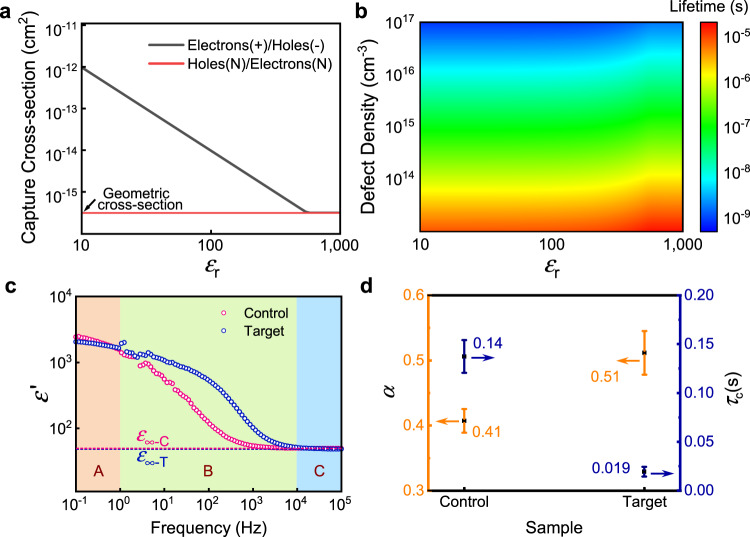
Defect-related dielectric properties in perovskite films. **a** Dielectric-dependent defect capture cross-sections of charge carriers. The electrical properties of the corresponding defects that capture the carriers are also shown. “Plus” means positively charged defects, “minus” means negatively charged defects and “N” represents neutral defects, respectively. **b** Impacts of dielectric constant and defect density on the carrier lifetime in the high-injection limit. **c** The real part of dielectric constant for perovskite films as a function of frequency: range A (low frequency), range B (moderate frequency), and range C (high frequency). **d** Fitting results of frequency-dependent dielectric constant for perovskite films based on the Cole-Cole equation, where parameter *τ*_c_ is the relaxation time constant, and *α* reflects the width of relaxation time distribution. The error bars stand for the confidence interval (CI) with 95% confidence.

To better understand the correlation between the defect capture cross-section and the dielectric constant, Fig. 1a presents the dielectric constant dependence of the capture cross-section of defects, and we note that higher dielectric constant is required to mitigate the capture cross-section of defects. Figure 1b shows the prediction of the carrier lifetimes with different defect densities and different dielectric constants in the high-injection limit. The detailed model based on the Shockley-Read-Hall (SRH) recombination theory^36^ is described in Supplementary Note 2. In general, for a given defect density, the photoexcited carrier lifetimes can be improved by increasing the dielectric constant.

In light of such a correlation, here we incorporate additional alkali halide species into the bication perovskites to regulate the dielectric responses. We determined the dielectric response of various bication perovskites by fabricating a sandwiched structure of ITO/Perovskite/Au. The complex dielectric constant ε* encompassing the real *ε*‘(*ω*) and imaginary *ε*“(*ω*) parts are obtained from impedance spectroscopy measurements^37,38^. Details of this method are described in Supplementary Note 3. In comparison to the film without any extrinsic alkali iodide, we found that adding sodium iodide (NaI) and rubidium iodide (RbI) did not effectively vary the frequency-dependent dielectric constant, while the addition of potassium iodide (KI) significantly influenced the dielectric response (Supplementary Fig. 1). This difference may stem from the different distributions of the alkali ions^34,39,40^. We further confirmed that the variation of the dielectric response was nearly independent of the halide ions (Supplementary Fig. 2). Hence, in the following section, we used KI to regulate the dielectric constant, wherein the bication perovskites without and with KI are marked as “control” and “target”, respectively.

Figure 1c and Supplementary Fig. 3 compare the real and imaginary parts of the dielectric response of control and target samples as a function of frequency. The real part of the dielectric constant can be divided into three regions (Fig. 1c): a low-frequency region (<1 Hz, marked as A), a moderate-frequency region (1–10^4^ Hz, marked as B), and a high-frequency region (>10^4^ Hz, marked as C). Notably, the target sample shows a remarkably higher dielectric response than the control in region B (here we termed as the acceleration of the dielectric response), whereas there are negligible changes in regions A and C. We find that the imaginary part of the dielectric constant *ε*“(*ω*) slightly deviates from the Cole-Cole equation, due to the effect of the DC conductance of the perovskite films^41^, but the real part of dielectric constant *ε*‘(*ω*) obeys it (for details see Supplementary Note 1). Thus, the real parts of the two samples are fit using Cole-Cole equation, and the results are shown in Fig. 1d. The higher α value is indicative of the much broader relaxation time distribution for the target sample in comparison to that of the control, suggesting that the extra KI-related dielectric response appears in the target sample. Moreover, the lower relaxation time constant (*τ*_c_) of the target sample than the control further demonstrates a faster response to the electric field.

**Fig. 2:**
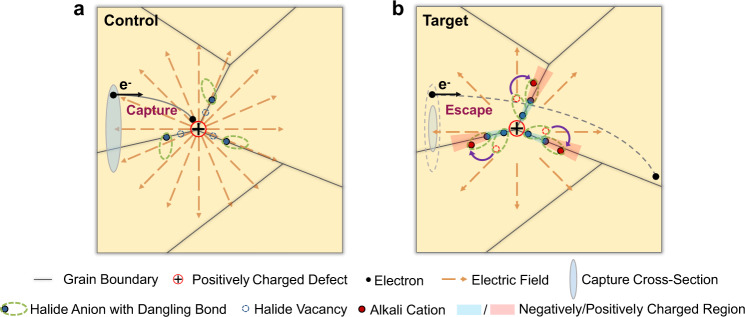
Carrier capture event by charged defects. **a**, **b** Schematic diagram of the defect capture process in the control (**a**) and target (**b**) perovskite films. Here we only show the process related to electrons for simplicity, and the same principle is also applicable to holes.

Based on these observations, we proposed an elaborate mechanism for the dielectric screening in the presence of the extrinsic potassium halide species. For the control perovskite films, grain boundaries are where most of the defects locate, including halide vacancies and halide dangling bonds as shown in Fig. 2a. When adding potassium halide species to the perovskite films, the added halides can fill the halide vacancies at the grain boundaries while potassium cations have the potential to couple with the halide dangling bonds. The potassium-based species are likely to redistribute in the presence of localized electric fields caused by charged defects^35^. Such behavior can significantly influence the spatial distribution and response mode of the space charge within perovskites and, therefore, should influence the dielectric response in the space-charge frequency region below 10^6^ Hz^24^, accounting for the difference observed in the region B. The negligible changes in regions A and C, which are contradictory to the dielectric enhancement triggered by extra ion migration^42^, further exclude the possibility of potassium-induced ion migration, as described in numerous studies that show a suppressed ion migration and hysteresis by the introduction of KI species^43,44^. The variation of space-charge distribution induced by the added potassium halide species can greatly reduce the defect capture cross-section of the charged defects located at the grain boundaries by the accelerated dielectric response, thereby effectively screening charged defects there. Such a dielectric-screening effect can mitigate charged defects, making them seem “invisible” to the charge carriers, i.e., defect stealth, which is beneficial to the charge-carrier transport within the films (Fig. 2b).

### Crystal structure and morphology

**Fig. 3:**
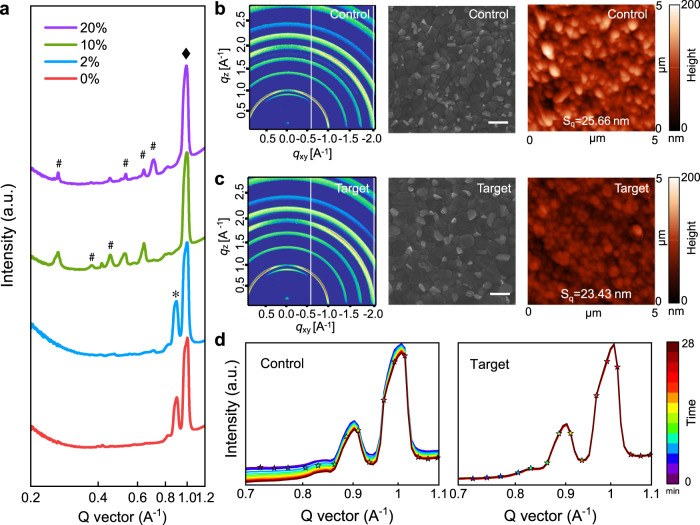
Crystal structure and morphology. **a** The grazing-incidence X-ray diffraction (GIXD) profiles of perovskite films with different K^+^ concentrations. The peaks labeled by “diamond”, “asterisk”, and “hash” are ascribed to bication perovskite, PbI_2_, and K_x_Pb_y_I_z_Br_(x+2y−z)_, respectively. **b**, **c** Grazing-incidence wide-angle X-ray scattering (GIWAXS) maps, top-view scanning electron microscopy (SEM) images and atomic force microscopy (AFM) images of the control (**b**) and target (**c**) perovskite films. Scale bar, 500 nm. **d** In situ time-resolved GIXD patterns under 530-nm light illumination of the control and target perovskite films in the characteristic Q vector region.

Following the dielectric study, we further performed structural, morphological and photophysical studies on a series of samples in the following section to fully elucidate the potential impacts of potassium halide species on the bication perovskites. Figure 3a shows the grazing-incidence X-ray diffraction (GIXD) profiles of perovskite films with different potassium concentrations, derived from the two-dimensional (2D) grazing-incidence wide-angle X-ray scattering (GIWAXS) patterns of those samples (Fig. 3b, c and Supplementary Fig. 4). The GIWAXS maps of the control and 2% K^+^-containing films present the same crystal structure and characteristic peaks at 1.0 Å^−1^ and 0.9 Å^−1^ being assigned to perovskite and PbI_2_^45^, respectively, owing to the use of the nonstoichiometric recipe with excess PbI_2_. Additionally, it is found that the peak of PbI_2_ disappeared and several small peaks emerged in the lower scattering vector *q* when the K^+^ concentration became higher. These are indexed to non-three- dimensional (non-3D) K_x_Pb_y_I_z_Br_(x+2y−z)_ perovskite phases, including non-perovskite KPb_2_X_5_^46,47^ and low-dimensional potassium-based perovskite phase K_2_PbX_4_^48^. These non-3D perovskite phases may behave as defects and imperfections, thus hampering the device performance improvement, as shown in Supplementary Table 1. It is also noted that a trace amount of potassium (2%) hardly changes the ultraviolet-visible (UV-Vis) absorption spectra for the perovskite films (identical bandgap ~1.62 eV) compared to the significantly shrunk bandgaps with higher K^+^ concentrations (see Supplementary Figs. 5a, b). Based on the GIXD and UV-vis absorption results, we used K^+^-containing (2%) perovskite as the target perovskite in our following studies.

In addition to the crystal structure, we also tracked the variation of the perovskite crystallinity under light illumination (Supplementary Fig. 6). A 530-nm green light (with intensity equivalent to 1 sun) was used to illuminate the control and target samples. In the case of the in situ light-soaking GIXD measurement, a highly stable diffraction pattern is observed for the target film (Fig. 3d). Although the peak position of the control film has no obvious shift during light soaking, the intensity of the peak gradually decreases. This variation is attributed to a decrease in the crystallinity, in contrast to the invariant GIXD patterns for target film under continuous light illumination, suggesting that the target sample can stabilize the crystal feature of the perovskite and exhibits enhanced photostability under the light illumination. From the scanning electron microscopy (SEM) images and the atomic force microscopy (AFM) topography images shown in Fig. 3b, c, we observe that the target film has a larger grain size relative to the control. At the same time, the smoother surface of the target film is evidenced by the AFM topography images both on PTAA (Fig. 3b, c) and ITO (Supplementary Fig. 7). The target sample also has a higher work function, as indicated by the Kelvin probe force microscopy (KPFM) (Supplementary Fig. 8), consistent with the ultraviolet photoelectron spectra (UPS) results (Supplementary Fig. 9). Compared to the control film, the Fermi level of the target film shifts towards the conduction band minimum (Supplementary Fig. 10), indicating a more n-type behavior for the target film. These improvements may be translated into significantly improved optoelectronic properties.

### Enhanced carrier lifetime and subdued surface recombination

**Fig. 4:**
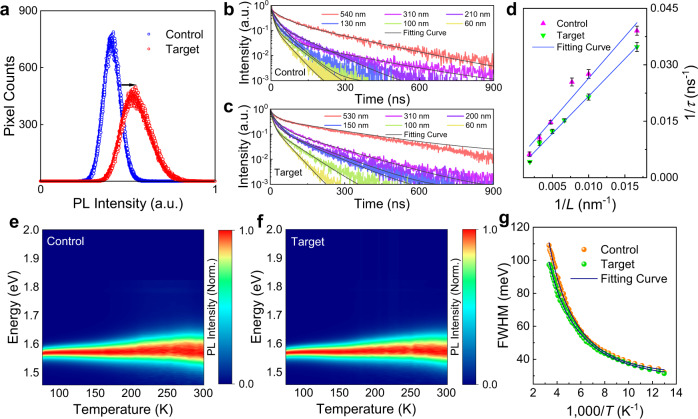
Photoluminescence and electron–phonon coupling. **a** Photoluminescence (PL) emission intensity distribution. **b**, **c** Time-resolved photoluminescence (TRPL) decays of the control (**b**) and target (**c**) samples versus different thicknesses. **d** The dependence of carrier lifetime on film thickness for the control and target samples and corresponding linear fitting plots. The error bars are the standard error (SE). **e**, **f** PL intensities mapped with the emission energy and temperature for the control (**e**) and target (**f**) films. **g** Temperature-dependent full width at half-maximum (FWHM) of PL emission peaks and the fitting curves based on independent Boson model.

To corroborate the improvement in optoelectronic properties of the target films by dielectric screening, with a particular focus on the reduction of nonradiative recombination losses, a series of photoluminescence (PL) measurements were performed to reveal the charge-carrier recombination processes. Figure 4a shows the PL intensity distribution over the sample surface under the same excitation intensity, which is extracted from the confocal microscopy PL maps shown in Supplementary Fig. 11. From these analyses, it can be seen that the target film shows a higher PL intensity, suggesting that nonradiative recombination pathways of photoexcited carriers are substantially suppressed in the target film, in agreement with the theoretical prediction. By measuring time-resolved photoluminescence (TRPL) decays of the samples consisting of ITO/PTAA/Perovskite (with varied thicknesses), we extract the carrier average lifetime by applying biexponential decay law for each decay curve (Fig. 4b, c). For thicknesses close to ~530 nm (used in the full device), the target film shows a longer lifetime in comparison to the control film. A remarkable increase in the carrier lifetime of the target film is consistent with the trends of the increased dielectric constant from the calculation shown in Fig. 1b.

Further analysis of the dependence of carrier lifetime on film thickness was performed to understand the surface recombination process. Considering the perovskite films are generally grown on the strain-containing substrates, unwanted defects and imperfections may be preferentially located at the interfaces between the perovskites and underlying charge transport layers. Among these defects and imperfections, mobile halide vacancies result in asymmetric energy-band bending, which accelerates the device sensitivity to the surface recombination between the perovskite and hole transport layer^49^. We also systematically quantified the surface recombination of the control and target films. In general, the carrier average lifetime *τ* has a simple dependence on bulk lifetime *τ*_B_, film thickness *L* and surface recombination velocity SRV: $\frac{1}{\tau }=\frac{1}{{\tau }_{\text{B}}}+\frac{\text{SRV}}{L}$ ^50,51^. Figure 4d shows plots of the 1/*τ* as a function of 1/*L*. The linear fits yield SRVs of the control and target perovskite films to be 222 ± 22 and 198 ± 7 cm s^−1^, respectively, indicating that the introduction of potassium halide species has reduced the surface recombination velocity. Furthermore, the longer bulk lifetime *τ*_B_ (549 ± 195 ns) of the target perovskite film (for the control film: 234 ± 106 ns) supports the argument that the target film also reduces the recombination rate in the bulk.

These results suggest that the adverse effects (low photoluminescence emission, short carrier lifetime, and inferior photostability) resulting from defect-assisted nonradiative recombination (subsists both in bulk and surface regions) are substantially improved with the use of target perovskites. Experimental observations and theoretical calculation collectively show that the performance improvement directly correlates with the accelerated dielectric response, which stimulate the dielectric-screening effect accompanying the reduction of the defect capture cross-section. In accordance with surface and cross-sectional energy-dispersive X-ray spectroscopy (EDS) mapping images (Supplementary Figs. 12 and 13) of the target perovskite film, we note that the potassium cations mainly situate at the grain boundaries distributed throughout the entire film rather than segregation to the interfaces. We also attempted to investigate the precise location of potassium cations within perovskite films by combining with the transmission electron microscopy (TEM) and X-ray diffraction (XRD), and the corresponding results are shown in Supplementary Figs. 14 and 15. From the TEM and XRD analyses, the smaller potassium cations are not incorporated into the perovskite lattice since no distinct change in the lattice spacing was observed, and are most likely to be at the grain boundaries, keeping with the variation of the dielectric response discussed above.

### Anomalous electron–phonon coupling reduction

With reduced nonradiative recombination losses, phonon scattering processes should be minimized as well^52^. The predominant electron–phonon coupling mechanism in metal halide perovskites is attributed to Fröhlich interaction between the longitudinal optical (LO) mode phonons and electrons. In this case, the dependence of the full width at half-maximum (FWHM) of the PL spectra on temperature [*Γ*(*T*)] can be described by the independent Boson model: $\varGamma (T)={\varGamma }_{0}+{\varGamma }_{\text{LO}}={\varGamma }_{0}+\frac{{\gamma }_{\text{LO}}}{{e}^{{E}_{\text{LO}}/{k}_{\text{B}}T}-1}$ ^53^, where *Γ*_0_ is the temperature-independent term induced by disorder and imperfections. *Γ*_LO_ stands for the contribution from LO phonons, and *γ*_LO_ is the coupling strength between electrons and LO phonons, *E*_LO_ is the representative energy of LO phonons described by Bose-Einstein distribution function. To elucidate this photophysical process, temperature-dependent PL spectra were measured over the temperature range of 80–300 K, as shown in Fig. 4e, f. The fit based on the Boson model is shown in Fig. 4g to compare the electron–phonon coupling property between the control and target perovskite films. Corresponding to the temperature-dependent FWHM, the target film has a lower electron–phonon coupling strength *γ*_LO_ of 85 ± 5 meV than 140 ± 8 meV of the control film. Typically, the enhanced intrinsic dielectric constant will lead to stronger electron–phonon coupling or polaronic effects^54–56^. However, the anomalously weaker electron–phonon coupling strength found in our experiment indicates that the increased dielectric constant is more likely from the contribution of the screening species localized at the grain boundaries rather than from the bulk of the perovskite grains, which further confirms the location of potassium halide species. As such, the higher effective dielectric constant induced by potassium halide species at the grain boundaries can screen the interaction between charge carriers and phonons from multiple grains and hinder the phonon emission, thus lowering scattering effects induced by phonons and further suppressing the nonradiative recombination losses.

### Device performance

**Fig. 5:**
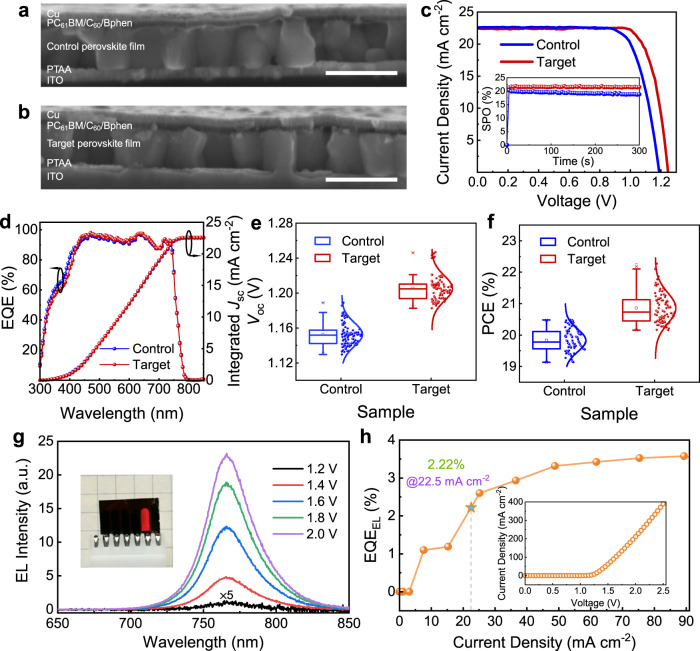
Photovoltaic and device characterization. **a**, **b** Cross-sectional scanning electron microscopy (SEM) images of the full device with the control (**a**) and target (**b**) perovskite films. Scale bar, 1 µm. **c** *J*–*V* characteristic of champion devices, and stabilized power output (SPO) of these cells at the maximum power point under simulated AM 1.5 G illumination (inset). **d** External quantum efficiency (EQE) spectra and the integrated short-circuit current density (*J*_sc_) for both the control and target perovskite photovoltaics (PPV) devices. **e**, **f** Corresponding photovoltaic merits with Gaussian fits of the open-circuit voltage (*V*_oc_) (**e**) and power conversion efficiency (PCE) (**f**) for 100 devices. The solid dots with the Gaussian fits represent original data. The boxplots denote the minimum and maximum data point (the lower and upper hyphen mark, respectively), the mean of the dataset (the square mark), and the 1st and the 99th percentiles (the lower and upper cross mark, respectively). Lower and upper boundaries of the boxes represent the 25th percentile and the 75th percentile, respectively. The lines within the boxes stand for the median of the dataset. The smallest and largest data point excluding outliers are given by the lower and upper whiskers, respectively. **g** Room-temperature electroluminescence (EL) spectra of the champion target PPV device under the different bias operating as a LED. Inset: the photograph of the target PPV device with a bias ~2 V. **h** The external quantum efficiency of the electroluminescence (EQE_EL_) as a function of the injected current density for the target PPV device. Inset: Current density versus the external voltage.

Finally, we evaluate the photovoltaic performance by fabricating the inverted PPV devices based on the two types of perovskite films. Compared to the control, the grains of the target films are much larger, even comparable to the thickness of perovskites used in the full device, as shown in Fig. 5a, b. We attained a power conversion efficiency of 22.3% [a fill factor (FF) of 0.80, a *V*_oc_ of 1.25 V, and a short-circuit current density (*J*_sc_) of 22.47 mA cm^−2^] for the champion device (Fig. 5c), in clear contrast with 20.5% of the control one (a FF of 0.76, a *V*_oc_ of 1.19 V, and a *J*_sc_ of 22.61 mA cm^−2^). Detailed photovoltaic parameters are listed in Supplementary Table 2. The champion target device was tracked at its maximum power point for 300 s and maintained its stabilized power conversion efficiency (~21.6%) with a negligible loss (inset in Fig. 5c), in agreement with the efficiency value obtained from the *J*–*V* curves. The *J*_sc_ (control: 22.53 mA cm^−2^, target: 22.58 mA cm^−2^) from external quantum efficiency (EQE) spectra (Fig. 5d) were consistent with the *J*_sc_ values from scanned *J*–*V* curves. In addition, the photostability of these fabricated cells were also characterized by continuous 1-sun illumination in a N_2_-filled glove box (Supplementary Fig. 16), and the device based on the target film shows a significantly superior stability under light illumination, while the efficiency of the control device showed a gradual decline during the continuous test. Substantial improvements and the excellent reproducibility of our results are evidenced in the parameter statistic boxplots in Fig. 5e, f and Supplementary Fig. 17. Remarkably, the average *V*_oc_ has improved more than 50 mV for target devices, and the record *V*_oc_ is up to 1.25 V for the perovskite with a 1.62 eV bandgap, yielding one of the lowest *V*_oc_ deficits of 0.37 V^4,34,57,58^.

To demonstrate the contributions to the voltage losses in the target device, we present a list of common losses in Table 1. A detailed calculation and explanation of each term is provided in Supplementary Note 5. According to the detailed balance theory, the open-circuit voltage in the radiative limit (${V}_{\text{oc},\text{rad}}$) is 1.34 V. Since there are nonradiative losses, the achieved *V*_oc_ is below the radiative limit. The *V*_oc_ loss, the gap between the voltage corresponding to the bandgap and achieved *V*_oc_, can be ascribed to: radiative (${\triangle V}_{\text{oc},\text{loss}}^{\text{rad}}$) and nonradiative losses (${\triangle V}_{\text{oc},\text{loss}}^{\text{nonrad}}$). In our cases, these are up to 280 mV and 100 mV, respectively. The nonradiative losses involve multiple processes including the losses from limited absorption (${\triangle V}_{\text{oc},\text{loss}1}$, 4 mV), the losses as actual device assembly (${\triangle V}_{\text{oc},\text{loss}2}$, 12 mV), and other losses during device operation (${\triangle V}_{\text{oc},\text{loss}3}$, 84 mV). As such, we could provide empirical expression to describe those losses: ${\triangle V}_{\text{oc},\text{loss}}^{\text{nonrad}}={\triangle V}_{\text{oc},\text{loss}1}+{\triangle V}_{\text{oc},\text{loss}2}+{\triangle V}_{\text{oc},\text{loss}3}$. In practice, the total nonradiative *V*_oc_ losses for a PPV device can also be quantified by the external quantum efficiency of the electroluminescence (EQE_EL_) when operating as a light-emitting diode (LED). We measured the electroluminescence emission of the target device, and Fig. 5g shows the EL spectra at different voltages, showing the dominant peak centered at 766 nm. The EQE_EL_ is measured to be 2.22% at a driving current that is equal to the short-circuit current (Fig. 5h). Following the approach in Supplementary Note 5^39^, we evaluated the nonradiative losses (${\text{Eva}.\triangle V}_{\text{oc},\text{loss}}^{\text{nonrad}}$) to be ~100 mV, which is in close agreement with the above analysis.

**Table 1:** A list of common voltage losses of the target PPV device.

	${\triangle V}_{{\rm{oc}},{\rm{loss}}}^{{\rm{rad}}}$	${\triangle V}_{{\rm{oc}},{\rm{loss}}}^{{\rm{nonrad}}}$	${\triangle V}_{{\rm{oc}},{\rm{loss}}1}$	${\triangle V}_{{\rm{oc}},{\rm{loss}}2}$	${\triangle V}_{{\rm{oc}},{\rm{loss}}3}$	${{\rm{Eva}}.\triangle V}_{{\rm{oc}},{\rm{loss}}}^{{\rm{nonrad}}}$
Calculations	$\frac{{E}_{{\rm{g}}}}{q}-{V}_{{\rm{oc}},{\rm{rad}}}$	${V}_{{\rm{oc}},{\rm{rad}}}-{V}_{{\rm{oc}}}$	${V}_{{\rm{oc}},{\rm{rad}}}-{V}_{{\rm{oc}},{\rm{rad}}}^{{\rm{film}}}$	${V}_{{\rm{oc}},{\rm{rad}}}^{{\rm{film}}}-{V}_{{\rm{oc}},{\rm{rad}}}^{{\rm{film}}-{\rm{to}}-{\rm{device}}}$	${V}_{{\rm{oc}},{\rm{rad}}}^{{\rm{film}}-{\rm{to}}-{\rm{device}}}-{V}_{{\rm{oc}}}$	$\frac{{k}_{{\rm{B}}}T}{q}{\rm{ln}}(\frac{1}{{{\rm{EQE}}}_{{\rm{EL}}}})$
Values (mV)	280	100	4	12	84	98

*E*_g_ bandgap of the perovskite film, *q* elementary charge, *V*_oc_ open-circuit voltage measured from *J*–*V* scan of the typical target device, *V*_oc,rad_ open-circuit voltage in the radiative limit, ${V}_{\text{oc},\text{rad}}^{\text{film}}$ theoretical *V*_oc_ calculated from the UV-vis absorption spectra (Supplementary Figs. 5, 18 and Note 4), ${V}_{\text{oc},\text{rad}}^{\text{film}-\text{to}-\text{device}}$ theoretical *V*_oc_ calculated from the measured photovoltaic EQE spectra (see in Fig. 5d and Supplementary Note 4), *k*_B_ Boltzmann constant, *T* temperature, EQE_EL_ measured external quantum efficiency of the electroluminescence for the device operating as a LED.

As a consequence, device performance enhancement has been shown to be related to the notable *V*_oc_ rise, which is a direct result of reduced nonradiative recombination losses. Multiple factors, including the dielectric-screening effect, optimized film quality, long-lived carrier lifetime, excellent interfacial extraction, decreased electron–phonon coupling strengths, reduced surface recombination velocities, and potassium-based defect passivation^34,59^, may be responsible for the mitigated nonradiative recombination losses, thus contributing to the device performance improvement. To quantify the contribution of those factors to the enhanced device performance, we assess their impacts on the *V*_oc_. In general, a high quality of the film (large-grained and better morphologies), longer carrier lifetime, weaker electron–phonon coupling and surface recombination, and defect passivation effect are strongly correlated with the reduced density of defects, which enables lower nonradiative losses and thus *V*_oc_ rise (Supplementary Note 6). In this regard, we employed dark space-charge-limited current (SCLC) measurements to determine the density of defects for control and target perovskites (Supplementary Fig. 19), by tracking a trap-filled limit voltage (*V*_TFL_) threshold of current–voltage curves for single-carrier devices, at which there exists 100% occupancy of available trap states via capturing carriers, so the extracted defect density, in this case, eliminates the influence of the dielectric-screening effect^60^. According to the calculation with the extracted defect density, we identify that the contribution to the *V*_oc_ improvement originating from the trap density reduction is less than 10.8 mV, which is much lower than the *V*_oc_ improvement of 60 mV in full devcies (Supplementary Table 1). The almost identical carrier mobility ~5.0 × 10^−3^ cm^2^ V^−1^ s^−1^ for both control and target films attained from SCLC measurements rules out the possibility that superior mobility being able to contribute to the *V*_oc_ rise. We further compare the carrier extraction capabilities at the perovskite-related interfaces (Supplementary Fig. 20), and derive that the target sample does not show significant benefits in terms of charge extraction at the contact interfaces, which evinces that the interfacial extraction in our devices is not critical for improving the *V*_oc_. Therefore, we reach the conclusion that the dielectric-screening-induced “defect stealth” dominates the inhibition of nonradiative losses within the full devices. In addition to mitigating the nonradiative recombination losses, the dielectric screening might also have a positive impact on the photocurrent response to light. In the case of low-intensity illumination condition, the charge transport and collection is predominantly relevant to the defect-related recombination that occurs within the perovskite absorbers^11^. We found that the PPV device fabricated with the target film has a much faster photocurrent response than that of the control device when illuminated by a 515-nm pulse light with low excitation intensity of ~3 mW cm^−2^ in Supplementary Fig. 21. The faster response time is most likely ascribed to the effective screening effect, facilitating the improved transport process of photogenerated charge carriers.

### Summary

In summary, we have demonstrated that the dielectric constant is a bridge connecting the Coulomb interaction of defects and phonons with charge carriers. The potassium halide species at the grain boundaries can reduce defect carrier capture cross-section as well as suppress electron–phonon coupling, which effectively screens the carrier nonradiative relaxation pathways. The accelerated dielectric response of the film not only greatly improves the optoelectronic properties of the perovskite but also boosts the power conversion efficiency to 22.3% for an inverted device, yielding a high *V*_oc_ of 1.25 V and a low voltage deficit of less than 0.37 V. Multiple characterization and calculation methods were applied to rationalize and confirm the observed low voltage deficit, and further quantitative analysis in the voltage loss provides a clear understanding of the different origins of nonradiative losses and corresponding contributions. This work provides a new paradigm to mitigate the adverse effect of defects from different angles, which will open a new avenue to further minimize nonradiative recombination losses in perovskite photovoltaics by dielectric regulation.

## Methods

### Materials and solvents

Lead diiodide (PbI_2_, 99.99%) and lead dibromide (PbBr_2_, 99%) were purchased from Tokyo Chemical Industry Co., Ltd. (TCI, Japan). Formamidinium iodide (FAI) was purchased from Dyesol (Australia). Cesium iodide (CsI, 99.999%) and potassium iodide (KI, ≥ 99.5%) were purchased from Sigma–Aldrich (USA). Sodium iodide (NaI, 99.999%) was purchased from Acros Organics (USA). Rubidium iodide (RbI, 99.8%) was purchased from Alfa Aesar (USA). Poly[bis(4-phenyl)(2,4,6-trimethylphenyl)amine] (PTAA) and bathophenanthroline (Bphen, >99%) were purchased from Xi’an Polymer Light Technology Corp. (China). Guanidinium bromide (GABr, >98%) was purchased from TCI (Japan). [6,6]-phenyl-C_61_-butyric acid methyl ester (PC_61_BM) was purchased from Nano-C Tech. (USA). 2,3,5,6-Tetrafluoro-7,7,8,8-tetracyanoquinodimethane (F_4_-TCNQ, 99%), and buckminsterfullerene (C_60_, 99%) were purchased from Jilin OLED Company (China). *N*,*N*-dimethylformamide (DMF, 99.8%), dimethyl sulfoxide (DMSO, 99.7%), isopropanol (IPA, 99.5%) and chlorobenzene (CB, 99.8%) were purchased from commercial sources (Acros Organics) and used as received. Toluene was purchased from Sinopharm Chemical Reagent Co., Ltd (China). Metal materials including gold (Au) and copper (Cu) were purchased from commercial sources with high purity (≥99.99%). All reagents were used as received without further purification.

### Perovskite precursor solution preparation

The control perovskite precursor solution was prepared by mixing PbI_2_ (1.08 M), FAI (1.12 M), PbBr_2_ (0.27 M) and CsI (0.17 M) in a mixed solvent of DMF/DMSO (4/1, v/v). For the perovskite precursor solution with *x*% K^+^, *x*% referred to c[K^+^]/(c[K^+^]+c[FA^+^]+c[Cs^+^]), and it was prepared by mixing PbI_2_ (1.08 M), FAI (1.12×(1 − *x*%) M), PbBr_2_ (0.27 M), CsI (0.17×(1 − *x*%) M), and KI (1.28 × *x*% M) in a mixed solvent of DMF/DMSO (4/1, v/v). The addition method of NaI and RbI is the same as that of KI described above. The prepared perovskite solution was stirred at 70 °C for 20 min before using. Doped-PTAA solution was prepared by adding F_4_-TCNQ solution (1 mg mL^−1^ in CB) into PTAA solution (10 mg mL^−1^ in toluene) with the weight ratio of F_4_-TCNQ being 1%. GABr solution was prepared by dissolving 5.5 mg of GABr into IPA (1 mL). PC_61_BM solution was prepared by dissolving 20 mg of PC_61_BM into CB (1 mL) and stirring at 70 °C for 2 h.

### Device fabrication

The inverted planar heterojunction PPV devices were fabricated on the prepatterned indium tin oxide (ITO) glass substrates. The prepatterned ITO glass substrates were ultrasonically cleaned with deionized water, acetone, diluted detergent, deionized water, acetone, IPA in succession for 20 min, followed by UV-ozone treatment for 10 min. The doped-PTAA solution was spin-coated on the as-cleaned ITO glass substrates at 4000 rpm for 30 s with a ramping rate of 1000 rpm s^−1^, and the samples were annealed at 150 °C for 10 min to form the hole transport layer. The perovskite precursor solution was spin-coated on hole transport layer by a two-step program: 2000 rpm for 10 s with a ramping rate of 200 rpm s^−1^, and 6000 rpm for 40 s with a ramping rate of 1000 rpm s^−1^. During the two-step spin-coating program, 100 μL of CB was poured on the center of the spinning substrate at 5 s prior to the end of the whole program. The samples were immediately annealed at 100 °C for 60 min to form the perovskite films. After cooling down to room temperature, 100 μL of GABr solution was spin-coated onto the perovskite films at 5000 rpm for 30 s with a ramping rate of 2500 rpm s^−1^, and the samples were annealed at 100 °C for 10 min to realize the solution-processed secondary growth of perovskite films. Then, 45 μL of PC_61_BM solution was spin-coated on the as-prepared perovskite films at 1000 rpm for 30 s with a ramping rate of 200 rpm s^−1^. After that, the samples were transferred to a vacuum chamber. C_60_ (20 nm) and Bphen (6 nm) were thermally evaporated in succession onto the samples in the vacuum chamber with the base pressure of <7 × 10^−5^ Pa. The metal copper electrode (85 nm) was evaporated in another vacuum chamber with the base pressure of <4 × 10^−4^ Pa. All of the above-mentioned spin-coating and annealing processes were conducted in a N_2_-filled glove box with H_2_O and O_2_ concentrations of < 0.1 ppm.

### PPV device measurements

The current density-voltage (*J*–*V*) characteristics of PPV devices were measured (2400 Series SourceMeter, Keithley Instruments) under simulated AM 1.5 G sunlight at 100 mW cm^−2^ irradiance by a 150 W class AAA solar simulator (XES-40S1, SAN-EI). The light intensity of 100 mW cm^−2^ was calibrated by using a standard monocrystalline silicon solar cell with a KG-5 filter. The *J*–*V* measurements were performed in a N_2_-filled glove box, with the *J*–*V* scan ranges from 1.22 V (−0.02 V) to −0.02 V (1.22 V) in reverse (forward) direction for the control PPV devices and from 1.25 V (−0.02 V) to −0.02 V (1.25 V) in reverse (forward) direction for the target PPV devices. Stabilized power output (SPO) was obtained by holding the devices at the fixed voltage of initial maximum power point under the simulated AM 1.5 G sunlight at 100 mW cm^−2^ recorded by a Keithley 2400 SourceMeter. For photostability testing, the unencapsulated devices were illuminated under continuous 100 mW cm^−2^ irradiation by the 150 W class AAA solar simulator (XES-40S1, SAN-EI) in a N_2_ atmosphere. The external quantum efficiencies (EQE) of the nonencapsulated inverted PSCs were acquired in air by a lock-in amplifier coupled with a monochromator (Crowntech, Qtest Station 2000 USA). A standard-monocrystalline silicon cell was used as the reference for the EQE tests. The external quantum efficiency of the electroluminescence (EQE_EL_) measurement was conducted using a Keithley 2400 source meter and a fibre integration sphere (CME-LP38) coupled with a spectrometer (Flame, Ocean Optics) at the room temperature (297 K) under the forward bias. The device was placed on top of the integration sphere and only forward light emission can be collected. Time-dependent photocurrent responses were characterized by an electrochemical workstation (Autolab PGSTAT302N, Metrohm, Switzerland) under monochromatic illumination with the incident light intensity of ~3 mW cm^−2^ generated by a 515-nm pulse light from a light-emitting diode (Huashang Laser Technology Co., Ltd.).

### Additional characterizations

The electrochemical impedance spectroscopy (EIS) was measured by an electrochemical workstation (Autolab PGSTAT302N, Metrohm, Switzerland). Grazing-incidence X-ray diffraction (GIXD) measurements were conducted on beamline 7.3.3 at Advanced Light Source, Lawrence Berkeley National Laboratory. The wavelength of X-ray was 1.240 Å with the grazing incident angle of 0.3 degree, and the scattering intensity was detected by a PILATUS 2 M detector. The exposure time was 5 s in single mode for each frame (2 min). The 2D grazing-incidence wide-angle X-ray scattering (GIWAXS) images were sector averaged using Nika software package. A 530-nm green light with intensity of ~25 mW cm^−2^ was used to illuminate the samples during the in situ light-soaking GIXD measurement. The scanning electron microscopy (SEM) images and energy-dispersive X-ray spectroscopy (EDS) analysis were obtained by a field-emission scanning electron microscope (FEI Nova Nano SEM 430). The atomic force microscopy (AFM) and Kelvin probe force microscopy (KPFM) images were obtained by an atomic force microscope (NTEGRA Solaris, NT-MDT). Confocal microscopy photoluminescence maps were measured by a laser scanning confocal microscope (A1Rsi, Nikon, Japan). The steady-state and time-resolved photoluminescence spectra (400-nm excitation and detected at 770 nm) and temperature-dependent photoluminescence spectra (excited by a 450-nm light beam) were measured by a fluorescence spectrophotometer (FLS1000, Edinburgh Instruments, England). The thicknesses of films were measured with a Dektak stylus profiler. The ultraviolet-visible (UV-vis) absorption spectra were measured by a spectrophotometer (UH4150, Hitachi, Japan). The ultraviolet photoelectron spectra (UPS) were recorded by an imaging photoelectron spectrometer (Axis Ultra, Kratos Analytical Ltd.) with a nonmonochromatic He Iα photon source (*hν* = 21.22 eV). Transmission electron microscopy (TEM, JEM-2100, JEOL) were used for characterization with an operating voltage at 200 kV. The point resolution is 2.3 Å and the line resolution is 1.4 Å, respectively. X-ray diffraction (XRD) experiment was performed by Mini Flex 600 (Rigaku, Japan). The current–voltage measurements for space-charge-limited current (SCLC) analysis were carried out with the Keithley 2400 SourceMeter under dark at the room temperature.

### Reporting summary

Further information on experimental design is available in the Nature Research Reporting Summary linked to this paper.

## Supplementary information

Supplementary Information

Solar Cells Reporting Summary

## Data Availability

The data that support the findings of this study are available from the corresponding author upon reasonable request.

## Code availability

All custom codes used to generate results in the current study are available from the corresponding author upon reasonable request.
