# Supplementary material for: Dielectric screening in perovskite photovoltaics
Source: Nat Commun. 2021 Apr 30;12:2479. doi: 10.1038/s41467-021-22783-z (PMC8087789; doi:10.1038/s41467-021-22783-z)
Supplement: Supplementary file 1 — Supplementary Information [file 41467_2021_22783_MOESM1_ESM.pdf]

Supplementary Information

Dielectric screening in perovskite photovoltaics

Rui Su[1†], Zhaojian Xu[1,2†], Jiang Wu[1†], Deying Luo[1,3], Qin Hu[4,5], Wenqiang Yang[1], Xiaoyu Yang[1], Ruopeng Zhang[6], Hongyu Yu[3], Thomas P. Russell[4,5], Qihuang Gong[1,7,8], Wei Zhang[9,10*] & Rui Zhu[1,7,8*]*

[1]State Key Laboratory for Artificial Microstructure and Mesoscopic Physics, School of Physics, Frontiers Science Center for Nano-optoelectronics & Collaborative Innovation Center of Quantum Matter, Peking University, Beijing 100871, China.

[2]Department of Electrical Engineering, Princeton University, Princeton, New Jersey 08544, USA.

[3]School of Microelectronics, Southern University of Science and Technology, Shenzhen 518055, China.

[4]Polymer Science and Engineering Department, University of Massachusetts, Amherst, Massachusetts 01003, USA.

[5]Materials Sciences Division, Lawrence Berkeley National Laboratory, Berkeley, California 94720, USA.

[6]National Center for Electron Microscopy, Molecular Foundry, Lawrence Berkeley National Laboratory, Berkeley, California 94720, USA.

[7]Collaborative Innovation Center of Extreme Optics, Shanxi University, Taiyuan 030006, China.

[8]Peking University Yangtze Delta Institute of Optoelectronics, Nantong, Jiangsu 226010, China.

[9]Advanced Technology Institute, University of Surrey, Guildford GU2 7XH, UK.

[10]State Centre for International Cooperation on Designer Low-Carbon and Environmental Material (SCICDLCEM), School of Materials Science and Engineering, Zhengzhou University, Zhengzhou 450001, China.

[†]These authors contributed equally: Rui Su, Zhaojian Xu, Jiang Wu.

[*]Correspondence and requests for materials should be addressed to R.Z. (email: iamzhurui@pku.edu.cn) or to W.Z. (email: wz0003@surrey.ac.uk) or to T.P.R. (email: tom.p.russell@gmail.com).

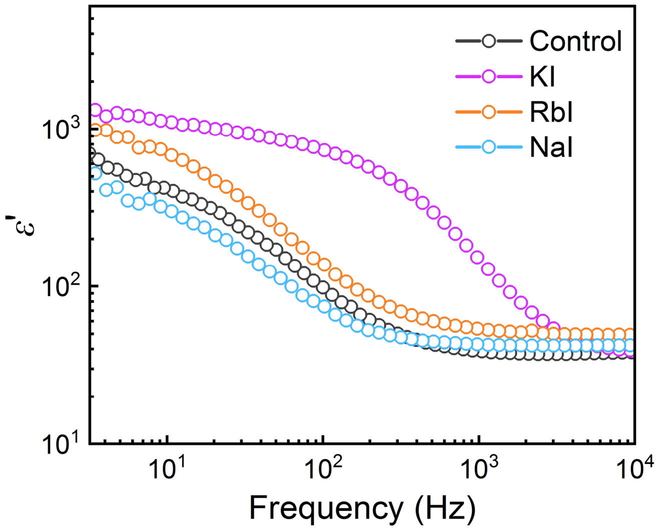

Supplementary Figure 1. Frequency-dependent dielectric constant (the real part) for perovskite films incorporating different alkali iodide species.

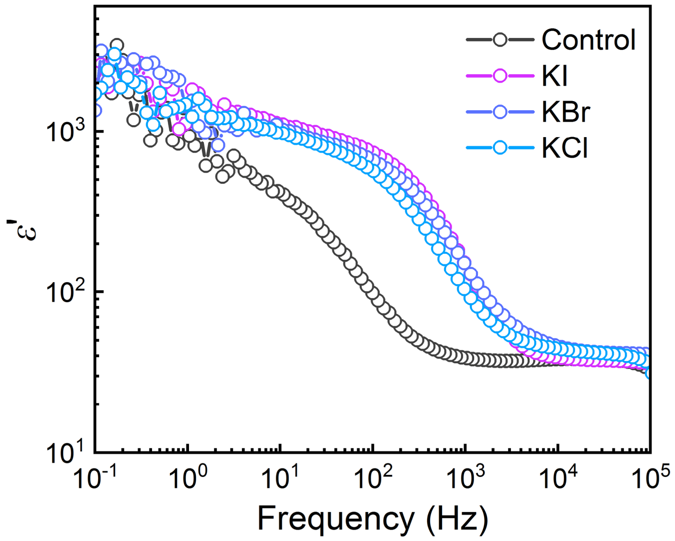

Supplementary Figure 2. Frequency-dependent dielectric constant (the real part) for perovskite films with addition of different potassium halide species.

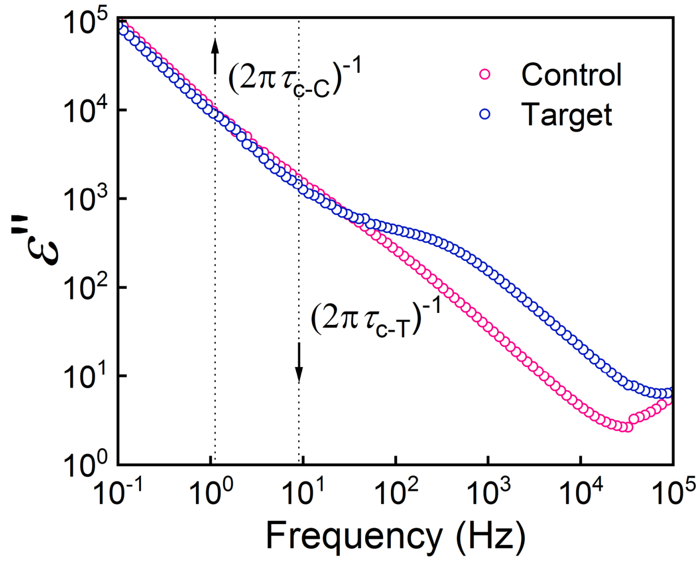

Supplementary Figure 3. Imaginary part of dielectric constant as a function of frequency for perovskite films. $\tau_{c\text{-}C}$ and $\tau_{c\text{-}T}$ represent the temperature-dependent constant (i.e., the relaxation time) of the control and target samples, respectively.

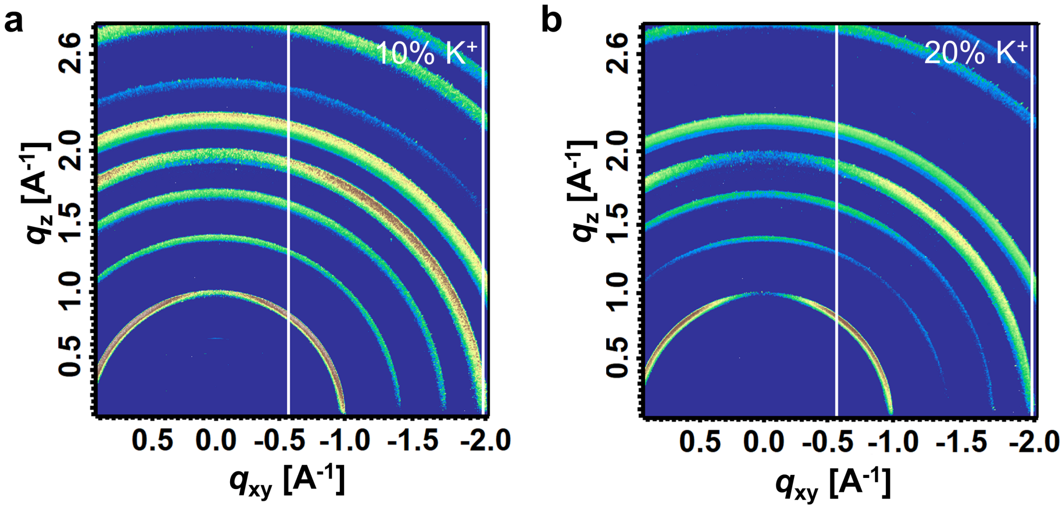

Supplementary Figure 4. a, **b** Two-dimensional (2D) grazing incidence wide-angle X-ray scattering (GIWAXS) images of the prepared target perovskite films with 10% (**a**) and 20% (**b**) K^+ concentrations.

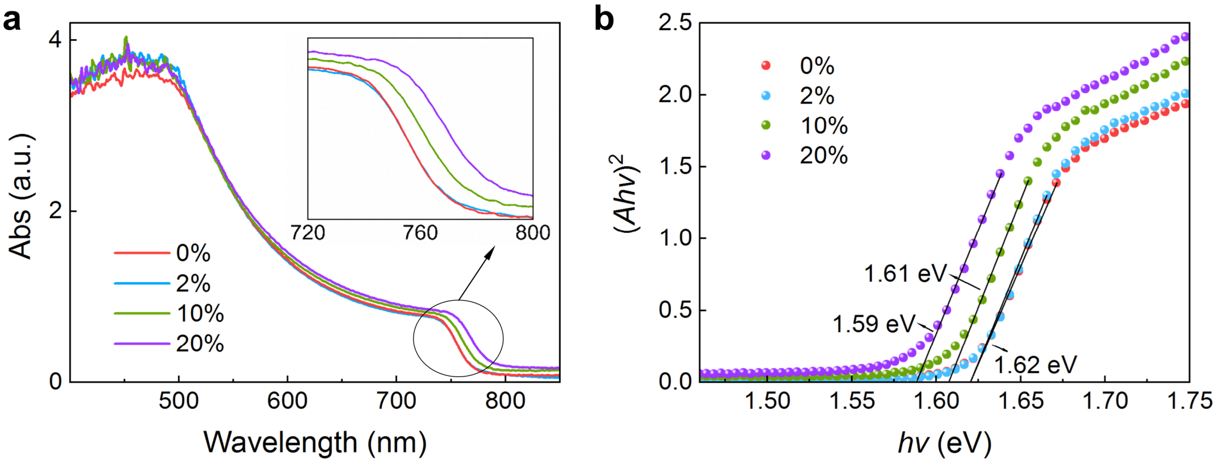

Supplementary Figure 5. a Ultraviolet-visible (UV-Vis) absorption spectra of perovskite films with various K^+ concentrations. The inset is an enlarged view of the absorption in the region of 720–800 nm. **b** Tauc plot and the corresponding optical bandgaps of perovskite films in panel **a**.

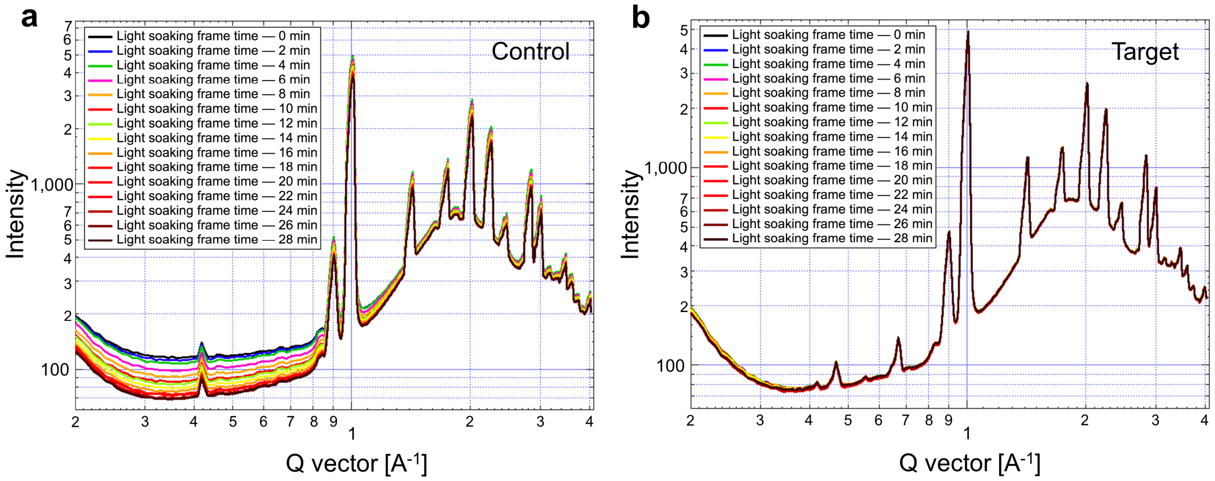

Supplementary Figure 6. a, **b** *In-situ* time-resolved grazing incidence X-ray diffraction (GIXD) profiles of the control (**a**) and target (**b**) perovskite films.

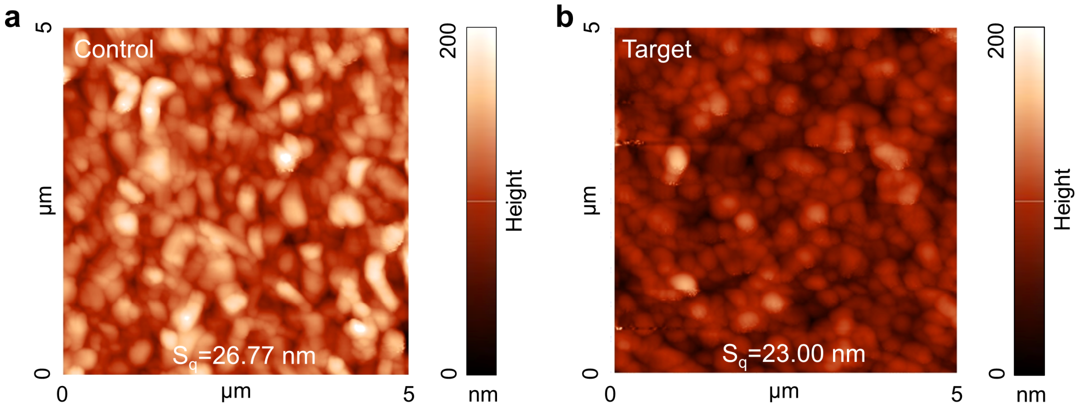

Supplementary Figure 7. a, **b** Atomic force microscopy (AFM) topography images of the control (**a**) and target (**b**) perovskite films on the ITO substrates.

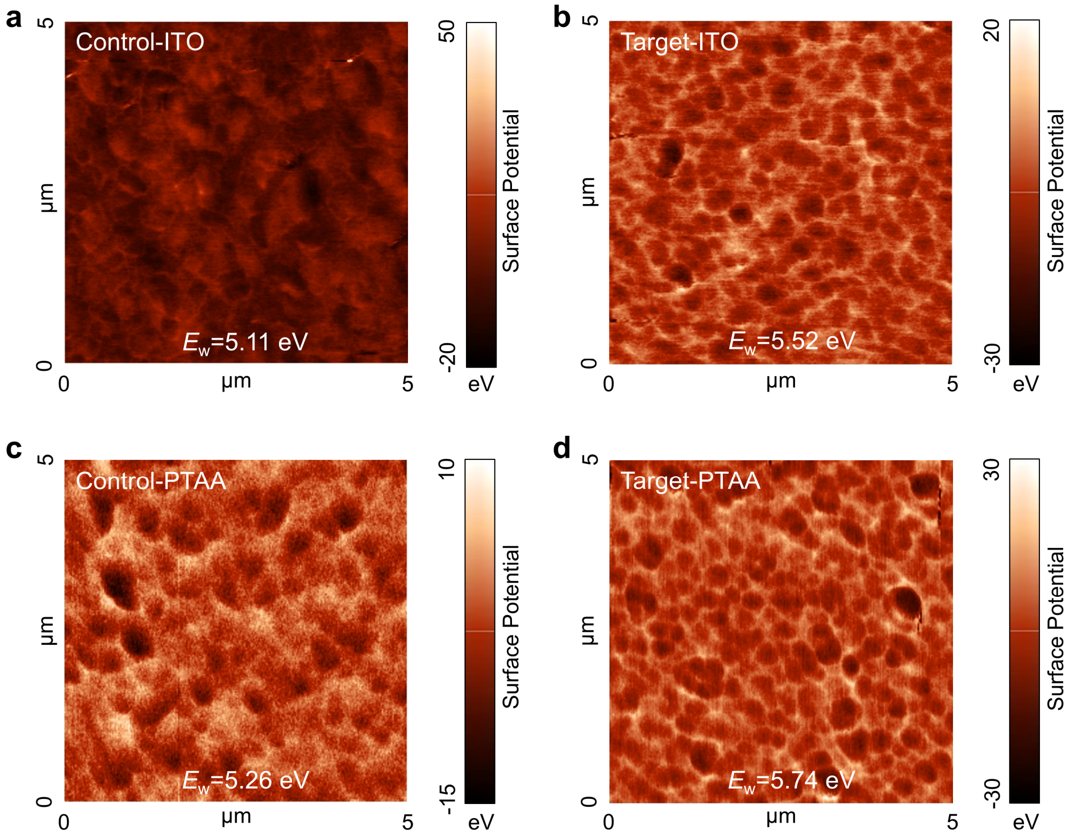

Supplementary Figure 8. a-d Kelvin probe force microscopy (KPFM) images of the control and target perovskite films on ITO substrates (**a**, **b**), and ITO/PTAA substrates (**c**, **d**).

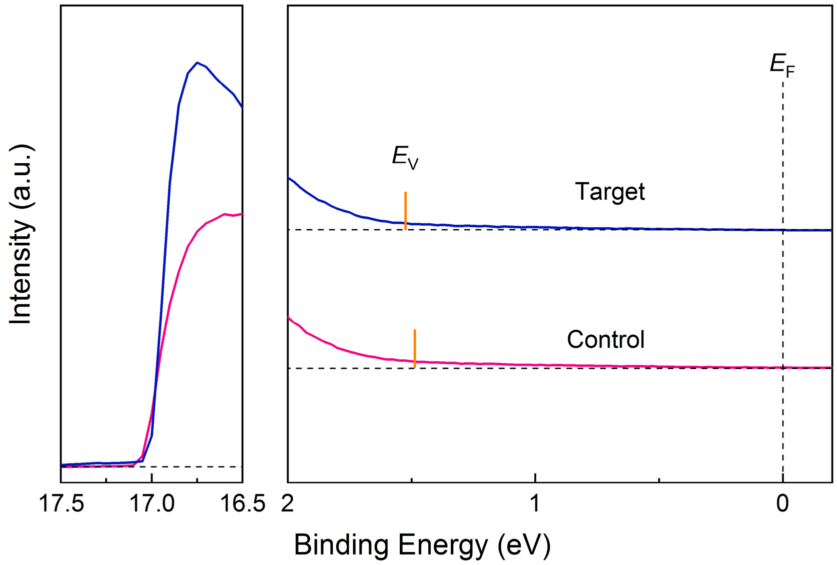

Supplementary Figure 9. Ultraviolet photoelectron spectra (UPS) of control and target perovskite films on ITO/PTAA substrates.

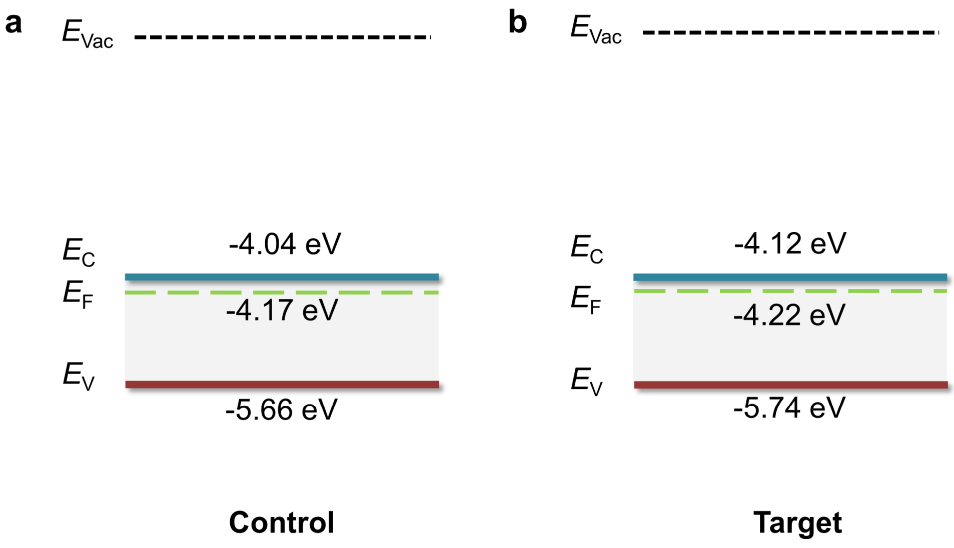

Supplementary Figure 10. a, **b** Schematic energy-level diagrams of the control (**a**) and target (**b**) perovskite films on ITO/PTAA substrates.

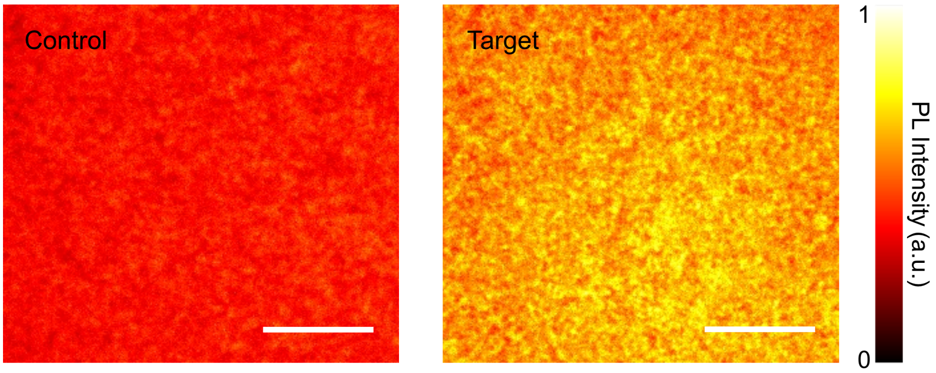

Supplementary Figure 11. Confocal photoluminescence (PL) maps of control and target films on ITO/PTAA substrates. Scale bar, 1μm.

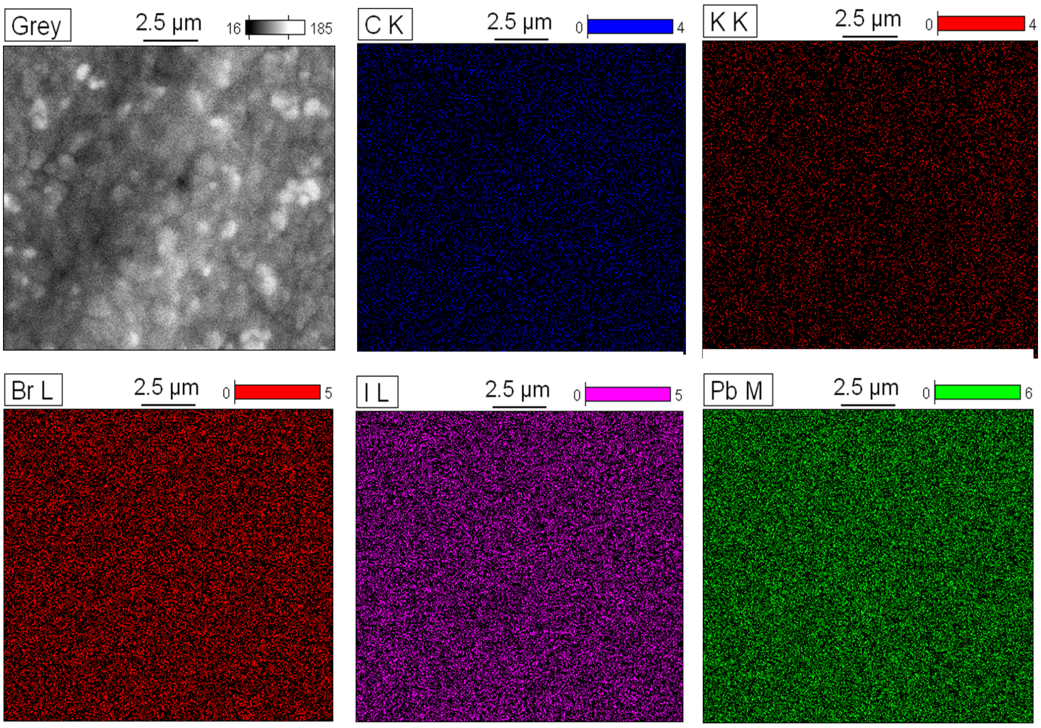

Supplementary Figure 12. Surface energy-dispersive X-ray spectroscopy (EDS) analysis of the target perovskite film.

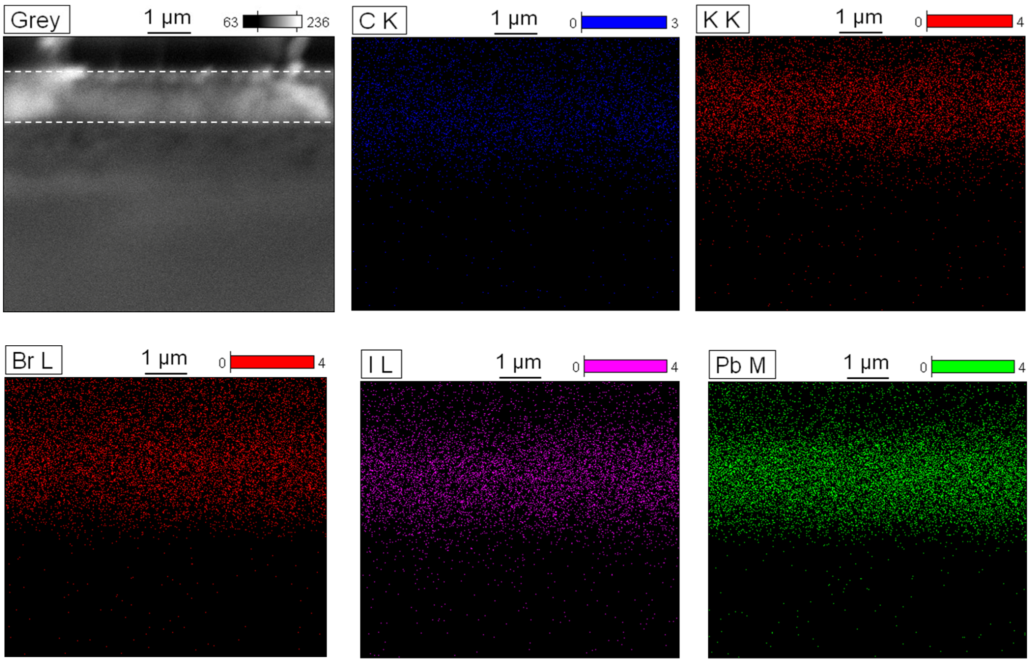

Supplementary Figure 13. Cross-sectional energy-dispersive X-ray spectroscopy (EDS) analysis of the target perovskite film.

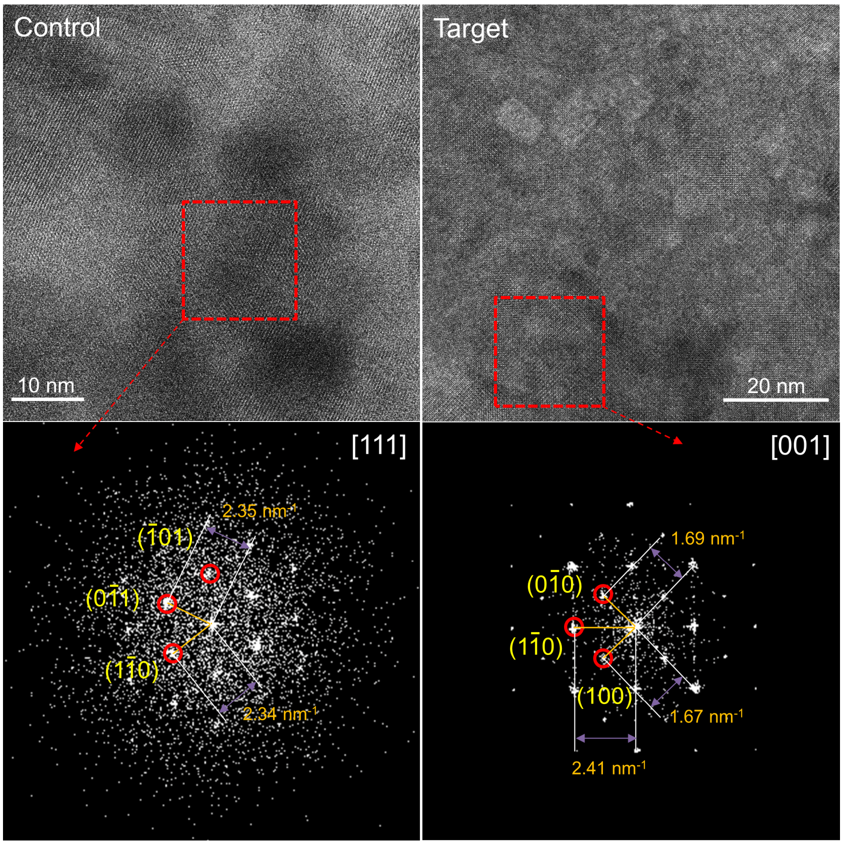

Supplementary Figure 14. Transmission electron microscopy (TEM) images and corresponding Fourier transforms of the control and target films.

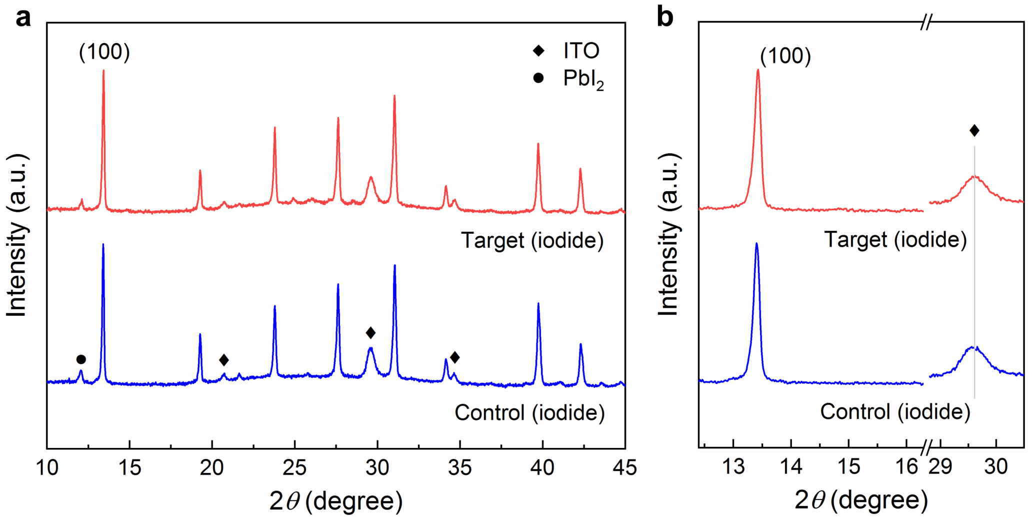

Supplementary Figure 15. a X-ray diffraction (XRD) patterns and **b** the zoom-in (100) peak of the XRD patterns of control and target perovskite films, the composition was chosen to be with same cation ratio but containing pure iodide as the anion site for comparison, excluding the influence of varied ratio of halide anions caused by the introduction of KI.

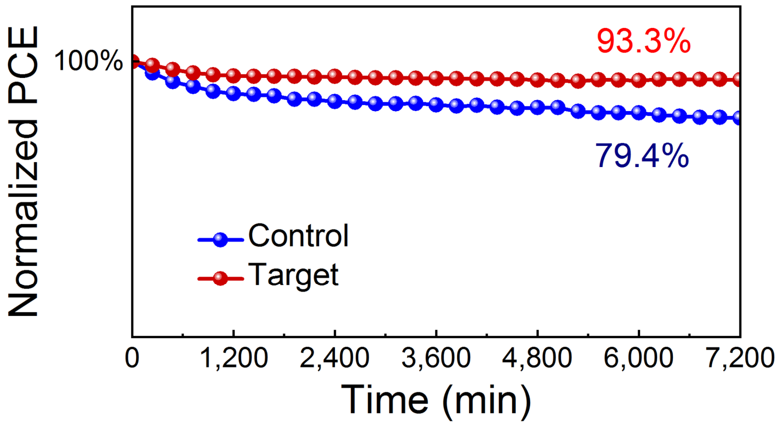

Supplementary Figure 16. Stability of the unencapsulated control and target devices under continuous 1-sun equivalent illumination in the N_2 atmosphere.

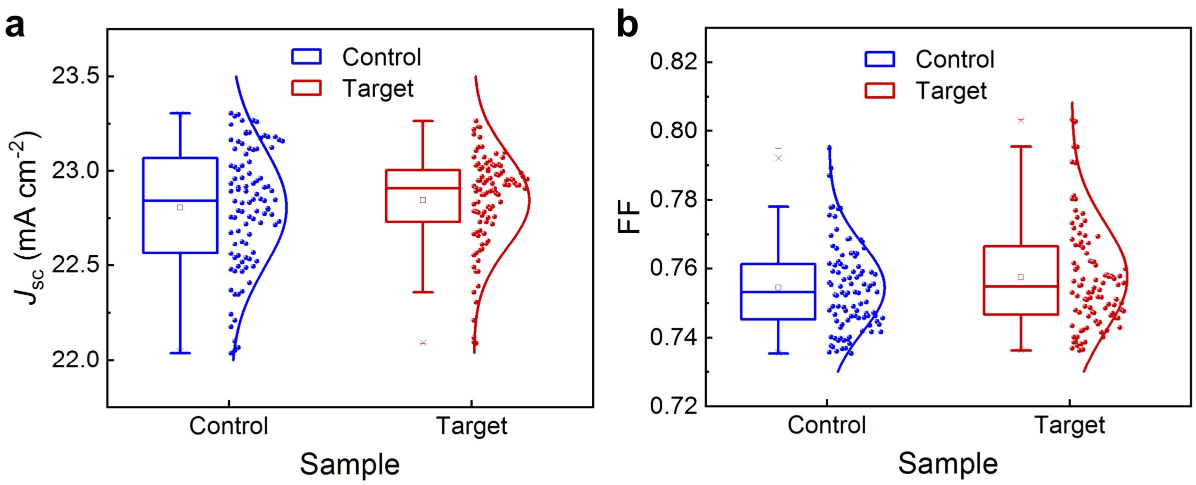

Supplementary Figure 17. a, **b** Statistic results with Gaussian fits of the short-circuit current density (J_{sc}) (**a**) and fill factor (FF) (**b**) for 100 devices. The solid dots with the Gaussian fits represent original data. The box plots denote the minimum and maximum data point (the lower and upper hyphen mark, respectively), the mean of the dataset (the square mark), and the 1st and the 99th percentiles (the lower and upper cross mark, respectively). Lower and upper boundaries of the boxes represent the 25th percentile and the 75th percentile, respectively. The lines within the boxes stand for the median of the dataset. The smallest and largest data point excluding outliers are given by the lower and upper whiskers, respectively.

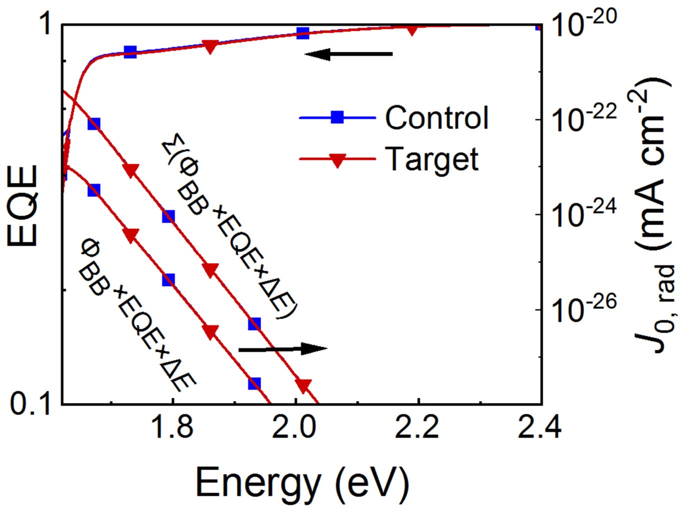

Supplementary Figure 18. Calculated external quantum efficiency (EQE) and dark saturation current density in the radiative limit from ultraviolet-visible (UV-vis) absorption spectra, giving the open-circuit voltage (V_{oc}) in the radiative limit of 1.3364 V (control) and 1.3359 V (target), respectively.

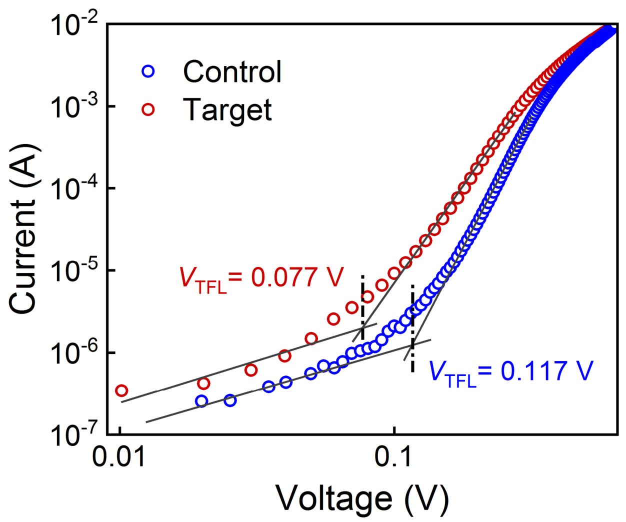

Supplementary Figure 19. Dark current-voltage curves of the electron-only devices (ITO/SnO_2/perovskite/$PC_{61}BM$/BCP/Cu) based on control and target films for space-charge-limited current (SCLC) model[1]. The densities of electron traps for the control and target samples are estimated to be 2.99×10^{15} cm^{-3} and 1.97×10^{15} cm^{-3}, respectively, according to the cross-over trap-filled limit voltage (V_{TFL}) that presented. In addition, the control and target perovskites give carrier mobilities of 5.01×10^{-3} cm^2 V^{-1} s^{-1} and 5.05×10^{-3} cm^2 V^{-1} s^{-1}, respectively, which is well fitted by the Mott–Gurney Law at the trap-filled SCLC regions.

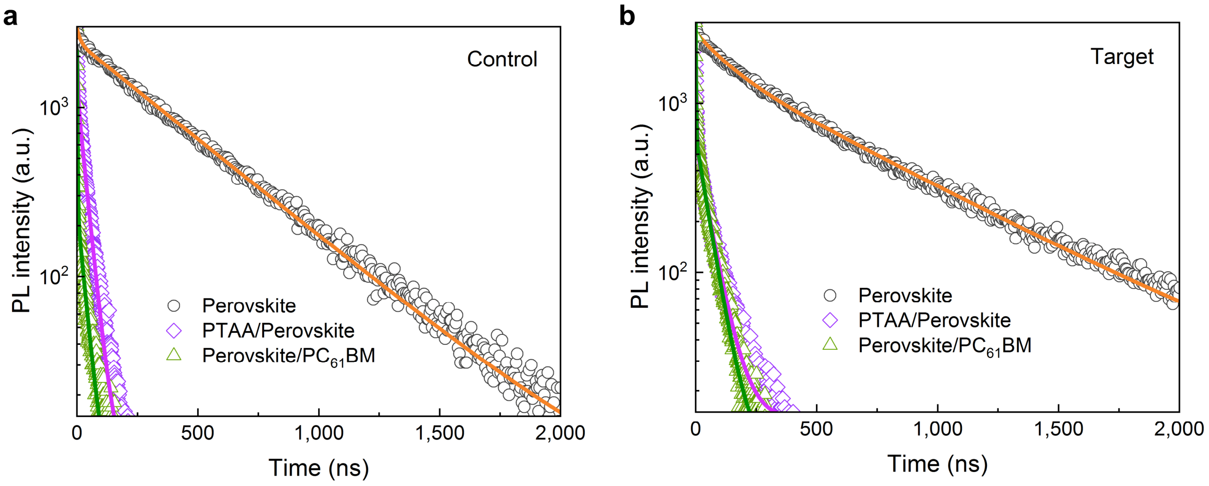

Supplementary Figure 20. a, b Time-resolved PL (TRPL) decays of the control (**a**) and target (**b**) samples with different film stack structures. The solid lines represent the fitting curves. When inserting $PC_{61}BM$ and PTAA layers, TRPL decays for both perovskites accelerate considerably under same excitation and collection conditions in comparison to the isolated films, suggesting a comparable charge extraction for those two samples.

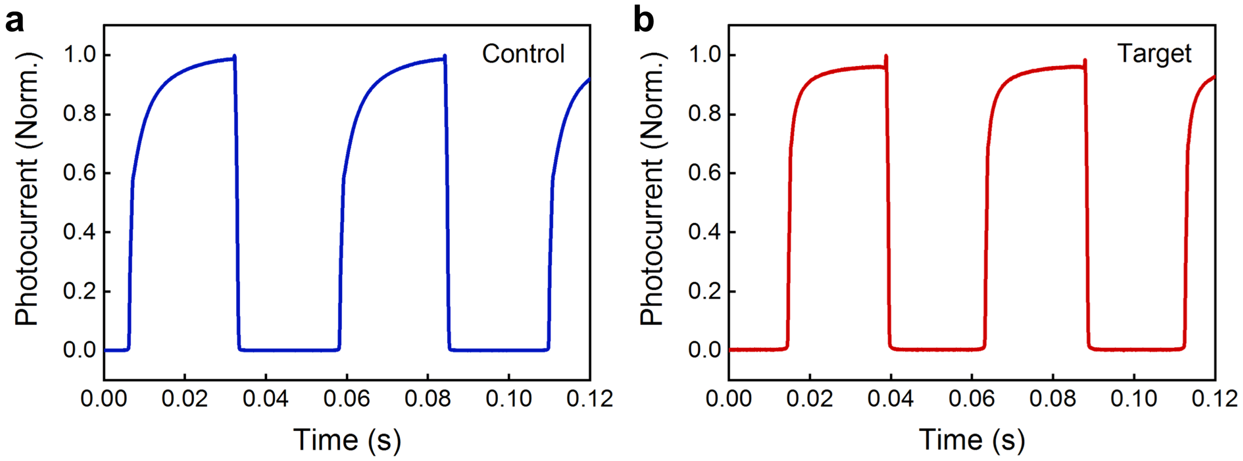

Supplementary Figure 21. a, b Time-dependent photocurrent responses of the control (**a**) and target (**b**) devices under the illumination condition of low excitation intensity (~3 mW cm^{-2}) by a 515-nm pulse light.

Supplementary Table 1. Comparison of the average performance for the inverted PPV devices processed with different K^+ concentration solutions from the statistical results. The error bars stand for the standard deviation (SD) of 10 devices (10%, 20%) and 100 devices (0%, 2%), respectively.

Samples	V_{oc} [V]	J_{sc} [mA cm^{-2}]	FF	PCE [%]
0%	1.15 ± 0.01	22.8 ± 0.3	0.75 ± 0.01	19.8 ± 0.4
2%	1.21 ± 0.01	22.8 ± 0.2	0.76 ± 0.02	20.9 ± 0.5
10%	1.01 ± 0.03	21.6 ± 0.9	0.52 ± 0.02	11.5 ± 0.9
20%	0.97 ± 0.03	20.9 ± 1.2	0.50 ± 0.04	10.1 ± 1.3

Supplementary Table 2. Summary of photovoltaic parameters for control and target inverted champion PPV devices.

Samples	Scan direction	V_{oc} [V]	J_{sc} [mA cm^{-2}]	FF	PCE [%]
Control	Reverse	1.189	22.61	0.762	20.5
	Forward	1.182	22.93	0.742	20.1
Target	Reverse	1.247	22.47	0.795	22.3
	Forward	1.241	22.83	0.776	22.0

Supplementary Note 1

Cole-Cole Equation

Cole-Cole equation is the most commonly used model to describe the dielectric relaxation in dielectric materials, and the general form is expressed as the following equation[2]:

$$\varepsilon^*(\omega)=\varepsilon_\infty+\frac{\varepsilon_s-\varepsilon_\infty}{1+(i\omega\tau_c)^{1-\alpha}}, \tag{1}$$

Where ε_∞ and ε_s are the "infinite frequency" and static dielectric constants. τ_c is the relaxation time constant, and α is the parameter reflecting the width of relaxation time distribution, which takes a value between 0 and 1[3], When $\alpha = 0$, there is only one single relaxation time, and the Cole-Cole equation becomes generic Debye expression. Supplementary Equation (1) can be further separated into real part:

$$\varepsilon'=\varepsilon_\infty+(\varepsilon_s-\varepsilon_\infty)\frac{1+(\omega\tau_c)^{1-\alpha}\sin(\alpha\pi/2)}{1+2(\omega\tau_c)^{1-\alpha}\sin(\alpha\pi/2)+(\omega\tau_c)^{2(1-\alpha)}} \tag{2}$$

and imaginary part:

$$\varepsilon''=(\varepsilon_s-\varepsilon_\infty)\frac{(\omega\tau_c)^{1-\alpha}\cos(\alpha\pi/2)}{1+2(\omega\tau_c)^{1-\alpha}\sin(\alpha\pi/2)+(\omega\tau_c)^{2(1-\alpha)}}. \tag{3}$$

Note that the Cole-Cole equation is applied to the ideal insulators and can describe their dielectric behavior. However, for the quasi-device applied in the impedance spectroscopy measurement, due to the effect of DC conductance of perovskite films, the sandwiched structure ITO/Perovskite/Au could be seen as an ideal dielectric capacitor connected in parallel with a resistance. What needs to be emphasized is the dielectric loss function $\varepsilon''(\omega)$ has a part of contribution from the perovskite intrinsic conductance, thus can't be fitted by Cole-Cole equation, and the relaxation peak of $\varepsilon''(\omega)$ could be covered by the conductance at the low frequency side as shown in Supplementary Fig. 3. Nevertheless, the real part of dielectric constant still obeys the Cole-Cole equation.

Supplementary Note 2

Calculation method for capture cross-section and carrier lifetime

The halide vacancy, due to its relatively low formation energy[4], is one of the main defects within perovskite films. Here, we mainly consider the influence of halide vacancy defects for simplicity. An ionized halide vacancy is positively charged and should capture an electron first before capturing a hole to finish the recombination. Considering the electron capture process, since electrons and ionized halide vacancies have opposite charges and have Coulomb attractive interactions, leading to larger capture cross-section than geometric cross-section of a halide atom. For the Coulomb attractive traps, we can set the Coulomb potential energy equal to the thermal energy to get the capture cross-section for electrons as[5]:

$$\sigma_{-} = \frac{q^4}{16\pi\left(\varepsilon_{\mathrm{r}}\varepsilon_0 k_{\mathrm{B}}T\right)^2}, \tag{4}$$

where q is the elementary charge, ε_r and ε_0 are dielectric constant and vacuum permittivity, respectively, k_B is the Boltzmann's constant and T is the temperature. We can obtain from the Supplementary Equation (4) that σ_- becomes smaller with dielectric constant increasing, which represents the dielectric screening effect. However, the σ_- should be always larger than the geometric cross-section of a halide atom, so a special condition should be pointed out: when the dielectric constant becomes so large that σ_- calculated from Supplementary Equation (4) is close to even lower than the geometric cross-section of a halide atom, Supplementary Equation (4) won't be reasonable anymore. An intuitive explanation is that dielectric constant originates from the collective response of a "bulk" of particles, which is a kind of mesoscopic property, and if the range of Coulomb interaction is down to the atom scale, the dielectric constant is not meaningful anymore. Accordingly, for extremely high dielectric constant, the screening effect is so strong that we can simply use geometric cross-section as the σ_-.

After the electron capture process, the trap becomes electrically neutral and can't produce long-range Coulomb potential under above circumstances, so the capture cross-section for holes,

of such a neutral trap is close to the geometric cross-section of a halide atom, which can be expressed as:

$$\sigma_{+} = \pi r^{2}, \tag{5}$$

where r is the radius of the halide atom, which is in the magnitude of 1 Å[6].

Typically, the net transition rate U for non-radiative recombination via defects, of density N_{t} and energy level E_{t}, can be described by Shockley-Read-Hall statistics as[7]:

$$U = \frac{\sigma_{+}\sigma_{-}v_{\mathrm{T+}}v_{\mathrm{T-}}N_{\mathrm{t}}\left(np - n_{\mathrm{i}}^{2}\right)}{\sigma_{-}v_{\mathrm{T-}}\left[n + n_{\mathrm{i}}\exp\left(\frac{E_{\mathrm{t}} - E_{\mathrm{i}}}{k_{\mathrm{B}}T}\right)\right] + \sigma_{+}v_{\mathrm{T+}}\left[p + n_{\mathrm{i}}\exp\left(\frac{E_{\mathrm{i}} - E_{\mathrm{t}}}{k_{\mathrm{B}}T}\right)\right]}, \tag{6}$$

where $v_{\mathrm{T+}}$ and $v_{\mathrm{T-}}$ are the thermal velocities related to the effective mass of holes m_{+}^{*} and electrons m_{-}^{*}, which are given by:

$$v_{\mathrm{T\pm}} = \sqrt{\frac{3k_{\mathrm{B}}T}{m_{\pm}^{*}}}, \tag{7}$$

n and p are the density of electrons and holes. n_{i} and E_{i} reflect the intrinsic carrier density and intrinsic Fermi level. k_{B} stands for the Boltzmann constant and T is the temperature. The injected carrier lifetime τ induced by Shockley-Read-Hall recombination, with the injected electrons and holes density of Δn and Δp (typically $\Delta n = \Delta p$), can be expressed as:

$$\tau = \frac{\Delta n}{U}. \tag{8}$$

Perovskite photovoltaic devices operating under AM 1.5G illumination is close to the high-injection limit where the photon-induced excess carriers are far greater than the carriers in equilibrium and the term containing the defect energy level can be ignored. Under this condition, the excess carrier lifetime can be simplified as:

$$\tau = \frac{1}{N_{\mathrm{t}}}\left(\frac{1}{v_{\mathrm{T+}}\sigma_{+}} + \frac{1}{v_{\mathrm{T-}}\sigma_{-}}\right) = \frac{1}{N_{\mathrm{t}}\sqrt{3k_{\mathrm{B}}T}}\left(\frac{\sqrt{m_{+}^{*}}}{\sigma_{+}} + \frac{\sqrt{m_{-}^{*}}}{\sigma_{-}}\right). \tag{9}$$

The effective mass of both electrons and holes are around 0.1 m_{e}[8, 9], where m_{e} is the mass of the free electron.

Supplementary Note 3

Extract complex dielectric constant ε* from impedance spectroscopy

For the sandwiched structure ITO/Perovskite/Au, we can see it as a parallel plate capacitor with complex dielectric constant ε^*. Thus, the real $\varepsilon'(\omega)$ and imaginary $\varepsilon''(\omega)$ parts of ε^* can be easily written as[10]:

$$\begin{aligned} \varepsilon' &= \frac{d}{\varepsilon_0 \omega A} \cdot \frac{Z''}{Z'^2 + Z''^2} \\ \varepsilon'' &= \frac{d}{\varepsilon_0 \omega A} \cdot \frac{Z'}{Z'^2 + Z''^2} \end{aligned}, \quad (10)$$

where ω stands for the measured angular frequency, d is the thickness of the perovskite film, A is the effective area of counter electrodes. Z' and Z'' are the real and imaginary parts of conjugate complex impendence from impedance spectroscopy, which is measured in dark at zero bias to minimize any electrochemical reactions at interfaces.

Supplementary Note 4

Calculation method for theoretical EQE and V_{oc} in the radiative limit

Assuming that each incident photon absorbed by the perovskite layer can produce one electron-hole pair and form the current, thus we can correspond the theoretical external quantum efficiency (EQE) to the absorption spectra in the following form:

$$\mathrm{EQE}(E) = 1 - 10^{-\mathrm{Abs}(E)}, \quad (11)$$

where Abs(E) represents the absorptance obtained from the ultraviolet-visible (UV-vis) absorption spectra.

Considering the radiative limit, when there non-radiative recombination does not exist, we can get the expression of dark saturation current density (J_0) and open-circuit voltage (V_{oc}) in the radiative limit[11, 12]:

$$\begin{aligned} J_{0,\mathrm{rad}} &= q\int \mathrm{EQE}(E)\cdot\Phi_{\mathrm{BB}}(E)\cdot dE \\ V_{\mathrm{oc,rad}} &= \frac{k_{\mathrm{B}}T}{q}\ln\left\{\frac{\int \mathrm{EQE}(E)\cdot\Phi_{\mathrm{AM\,1.5G}}(E)\cdot dE}{\int \mathrm{EQE}(E)\cdot\Phi_{\mathrm{BB}}(E)\cdot dE}+1\right\} \end{aligned}, \quad (12)$$

where k_B is the Boltzmann constant and T is the environment temperature. q represents the elementary charge. $\Phi_{AM\ 1.5G}$ and Φ_{BB} are the photon flux of the AM 1.5G standard solar spectrum and black body at 300 K respectively.

Supplementary Note 5

Evaluation of non-radiative losses—quantified by voltage losses

For an ideal solar cell, the maximum open-circuit voltage can be determined according to the detailed balance theory when only theoretically unavoidable loss processes (mainly radiative recombination) are present. In that case, the solar cell operates at its radiative limit and the corresponding open-circuit voltage is named as $V_{oc,rad}$. For our perovskite with a 1.62 eV bandgap, $V_{oc,rad}$ is 1.34 V[13]. Note that there is a voltage gap between the bandgap (V_g=E_g/q) and the $V_{oc,rad}$ of about 0.3 V for most perovskite solar cells. However, in reality, non-radiative losses are present and can be estimated by

$$\Delta V_{oc,loss}^{nonrad} = V_{oc,rad} - V_{oc} = \frac{k_B T}{q}\ln(\frac{1}{EQE_{EL}}), \quad (13)$$

where EQE_{EL} is the external quantum efficiency of electroluminescence for the solar cells. For the target device, we can estimate the voltage loss to be 98 mV by EQE_{EL} results.

From another perspective, the source of voltage losses includes the following parts:

a) Film losses—evaluated from the absorption spectra of the films ($V_{oc,rad} - V_{oc,rad}^{film}$).

We have predicted the voltage in the radiative limit based on the theoretical EQE of the individual perovskite films (named as $V_{oc,rad}^{film}$) in Supplementary Fig. 18. The theoretical EQE can be corresponded to the measured UV-vis absorption spectra of the films under the assumption that each incident photon absorbed by the perovskite layer can produce one electron-hole pair and form the current based on the method described in Supplementary Note 4. In this case, the target sample has a $V_{oc,rad}^{film}$ of ~1.336 V, showing a voltage gap estimated to be 4 mV with the $V_{oc,\ rad}$ of 1.34 V, which originates from the limited light absorption of the films.

b) Film-to-device losses—evaluated from the measured EQE of the devices ($V_{\mathrm{oc,rad}}^{\mathrm{film}} - V_{\mathrm{oc,rad}}^{\mathrm{film\text{-}to\text{-}device}}$).

In order to obtain more accurate voltage losses of the devices closer to the real working condition, we measured the photovoltaic EQE spectra (EQE_{PV}) of the target solar cell and evaluated the theoretical voltage ($V_{\mathrm{oc,rad}}^{\mathrm{film\text{-}to\text{-}device}}$) to be 1.324 V. Compared with the $V_{\mathrm{oc,rad}}^{\mathrm{film}}$ (1.336 V), there is another voltage gap of 12 mV, directly evidencing the voltage losses occur during the assembly process of the device, which is mainly caused by the non-unity internal quantum efficiency (IQE).

c) Device losses—evaluated from the *J–V* curves of the devices ($V_{\mathrm{oc,rad}}^{\mathrm{film\text{-}to\text{-}device}} - V_{\mathrm{oc}}$).

The V_{oc} of the target device used for EQE_{PV} measurement is 1.24 V (by *J–V* scan), resulting in the voltage gap of 84 mV ($V_{\mathrm{oc,rad}}^{\mathrm{film\text{-}to\text{-}device}} - V_{\mathrm{oc}}$) with the theoretical voltage attained by EQE_{PV}. This voltage gap is mainly due to the non-radiative losses within the solar cell device.

So far, a clear understanding of the voltage loss has basically been formed: the voltage losses include a) the loss of film absorption (4 mV), b) the loss during device assembly (12 mV) and c) other non-radiative losses during device operation (84 mV). All these three loss terms add up and equal to the non-radiative voltage losses (100 mV), which is in close agreement with the value estimated from the measured EQE_{EL} (98 mV):

$$\Delta V_{\mathrm{oc,loss}}^{\mathrm{nonrad}} = V_{\mathrm{oc,rad}} - V_{\mathrm{oc}} = \Delta V_{\mathrm{oc,loss1}} + \Delta V_{\mathrm{oc,loss2}} + \Delta V_{\mathrm{oc,loss3}}$$

$$\Delta V_{\mathrm{oc,loss1}} = V_{\mathrm{oc,rad}} - V_{\mathrm{oc,rad}}^{\mathrm{film}}$$

$$\Delta V_{\mathrm{oc,loss2}} = V_{\mathrm{oc,rad}}^{\mathrm{film}} - V_{\mathrm{oc,rad}}^{\mathrm{film\text{-}to\text{-}device}}$$

$$\Delta V_{\mathrm{oc,loss3}} = V_{\mathrm{oc,rad}}^{\mathrm{film\text{-}to\text{-}device}} - V_{\mathrm{oc}} \,. \tag{14}$$

Supplementary Note 6

Estimation of the contribution to enhanced V_{oc} from reduced defect density

Typically, the whole recombination process in the perovskite materials can be described as:

$$\frac{\mathrm{d}n}{\mathrm{d}t} = -k_1 n - k_2 n^2 - k_3 n^3 , \tag{15}$$

where k_1, k_2 and k_3 are the first-order, the second-order and the third-order recombination rate constants associated with defect-assisted, radiative and Auger recombination processes, respectively, and n is the photo-generated carrier density. The lower defect density mainly contributes to the reduced defect-assisted first-order recombination rate constant (k_1) with k_2 and k_3 basically unchanged, thus leading to suppressed non-radiative recombination loss.

If we assume that the suppressed non-radiative recombination loss only comes from the reduced defect density, then according to the SRH recombination theory[7], k_1 is approximately proportional to the defect density, so we have:

$$\frac{k_{1,\text{ Control}}}{k_{1,\text{ Target}}} \approx \frac{2.99\times10^{15}}{1.97\times10^{15}} = 1.52 . \tag{16}$$

The reduced defect density from 2.99 × 10^{15} cm^{-3} for control sample to 1.97 × 10^{15} cm^{-3} for target sample was observed in Supplementary Fig. 19.

The photoluminescence quantum yield (PLQY) relates to the recombination rate constants as:

$$\text{PLQY} = \frac{k_2 n}{k_1 + k_2 n}, \tag{17}$$

and the ratio of PLQY between control and target sample is given by:

$$\frac{\text{PLQY}_{\text{Target}}}{\text{PLQY}_{\text{Control}}} = \frac{k_{1,\text{ Control}} + k_2 n}{k_{1,\text{ Target}} + k_2 n} < \frac{k_{1,\text{ Control}}}{k_{1,\text{ Target}}} \approx 1.52 . \tag{18}$$

The V_{oc} loss resulting from non-radiative recombination can be described as[13]:

$$V_{\text{oc, loss}} = -\frac{k_B T}{q} \ln\left(\text{PLQY}\right), \tag{19}$$

where k_B is the Boltzmann's constant, T is the temperature and q is the elementary charge. Thus, the improvement of V_{oc} (or the reduction in the V_{oc} loss) from reduced defect density is calculated by:

$$\Delta V_{oc} = V_{oc,\,loss}^{Control} - V_{oc,\,loss}^{Target} = \frac{k_B T}{q} \ln\left(\frac{PLQY_{Target}}{PLQY_{Control}}\right) < \frac{k_B T}{q} \ln(1.52) \approx 0.42 \frac{k_B T}{q}. \quad (20)$$

Considering the room temperature, where T = 300 K, we can finally estimate the contribution to enhanced V_{oc} from reduced defect density:

$$\Delta V_{oc} < 10.8 \text{ mV}. \quad (21)$$

Supplementary References

1. Ren, H. et al. Efficient and stable Ruddlesden–Popper perovskite solar cell with tailored interlayer molecular interaction. *Nat. Photon.* **14**, 154–163 (2020).
2. Cole, K. S. & Cole, R. H. Dispersion and absorption in dielectrics I. Alternating current characteristics. *J. Chem. Phys.* **9**, 341–351 (1941).
3. Barsoukov, E. & Macdonald, J. R. *Impedance Spectroscopy: Theory, Experiment, and Applications* (John Wiley & Sons, Hoboken, 2005).
4. Yin, W.-J., Shi, T. & Yan, Y. Unusual defect physics in $CH_3NH_3PbI_3$ perovskite solar cell absorber. *Appl. Phys. Lett.* **104**, 063903 (2014).
5. Bube, R. H. *Photoelectronic Properties of Semiconductors* (Cambridge University Press, Cambridge, 1992).
6. Slater, J. C. Atomic radii in crystals. *J. Chem. Phys.* **41**, 3199–3204 (1964).
7. Sze, S. M. & Ng, K. K. *Physics of Semiconductor Devices* (John Wiley & Sons, Hoboken, 2006).
8. Brenner, T. M., Egger, D. A., Kronik, L., Hodes, G. & Cahen, D. Hybrid organic—inorganic perovskites: low-cost semiconductors with intriguing charge-transport properties. *Nat. Rev. Mater.* **1**, 15007 (2016).

9. Brivio, F., Butler, K. T., Walsh, A. & van Schilfgaarde, M. Relativistic quasiparticle self-consistent electronic structure of hybrid halide perovskite photovoltaic absorbers. *Phys. Rev. B* **89**, 155204 (2014).

10. Joshi, J. H., Kanchan, D. K., Joshi, M. J., Jethva, H. O. & Parikh, K. D. Dielectric relaxation, complex impedance and modulus spectroscopic studies of mix phase rod like cobalt sulfide nanoparticles. *Mater. Res. Bull.* **93**, 63–73 (2017).

11. Rau, U. Reciprocity relation between photovoltaic quantum efficiency and electroluminescent emission of solar cells. *Phys. Rev. B* **76**, 085303 (2007).

12. Luo, D. et al. Enhanced photovoltage for inverted planar heterojunction perovskite solar cells. *Science* **360**, 1442–1446 (2018).

13. Luo, D., Su, R., Zhang, W., Gong, Q. & Zhu, R. Minimizing non-radiative recombination losses in perovskite solar cells. *Nat. Rev. Mater.* **5**, 44–60 (2020).
