# Supplementary material for: Dielectric screening in perovskite photovoltaics
Source: Nat Commun. 2021 Apr 30;12:2479. doi: 10.1038/s41467-021-22783-z (PMC8087789; doi:10.1038/s41467-021-22783-z)
Supplement: Supplementary file 2 — Solar Cells Reporting Summary [file 41467_2021_22783_MOESM2_ESM.pdf]

nature research

Corresponding author(s): Prof. Rui Zhu
Dr. Wei Zhang
Prof. Thomas P. Russell

Solar Cells Reporting Summary

Nature Research wishes to improve the reproducibility of the work that we publish. This form is intended for publication with all accepted papers reporting the characterization of photovoltaic devices and provides structure for consistency and transparency in reporting. Some list items might not apply to an individual manuscript, but all fields must be completed for clarity.

For further information on Nature Research policies, including our data availability policy, see Authors & Referees.

Experimental design

Please check: are the following details reported in the manuscript?

1. Dimensions

Area of the tested solar cells
☒ Yes ☐ No
0.103 cm2

Method used to determine the device area
☒ Yes ☐ No
We employed a metal mask with 0.103 cm2 to define the device area.

2. Current-voltage characterization

Current density-voltage (J-V) plots in both forward and backward direction
☒ Yes ☐ No
The J-V curves were scanned in the reverse direction unless specified otherwise. The J-V photovoltaic parameters for the hysteresis study are obtained from scans in both forward and reverse directions. The information can be found in Methods-PPV device measurements and the plot is shown in Fig. 5c.

Voltage scan conditions
For instance: scan direction, speed, dwell times
☒ Yes ☐ No
The voltage scans were done in the reverse direction unless specified otherwise. Both forward and reverse scans were applied in the hysteresis study. Dwell time was not applied during the measurements. The information is stated in Methods-PPV device measurements.

Test environment
For instance: characterization temperature, in air or in glove box
☒ Yes ☐ No
We performed the device measurements in a N2-filled glove box at room temperature.

Protocol for preconditioning of the device before its characterization
☐ Yes ☒ No
No preconditioning of the device before its characterization.

Stability of the J-V characteristic
Verified with time evolution of the maximum power point or with the photocurrent at maximum power point; see ref. 7 for details.
☒ Yes ☐ No
We showed the stabilized power output (SPO) data in Fig. 5c (inset) determined by holding the cell at a fixed voltage near the maximum power point (MPP) on the J–V characteristic for 300 s.

3. Hysteresis or any other unusual behaviour

Description of the unusual behaviour observed during the characterization
☐ Yes ☒ No
There is no obvious hysteresis behavior observed for our devices.

Related experimental data
☒ Yes ☐ No
We showed both the forward and reverse scanned J-V photovoltaic parameters in Supplementary Table 2.

4. Efficiency

External quantum efficiency (EQE) or incident photons to current efficiency (IPCE)
☒ Yes ☐ No
We provided the EQE data in Fig. 5d. Details are given in Methods-PPV device measurements.

A comparison between the integrated response under the standard reference spectrum and the response measure under the simulator
☒ Yes ☐ No
The measured Jsc values from the J-V curves under AM 1.5G solar irradiation were consistent with the integrated short-circuit current densities from EQE spectra. This is stated in the device performance discussion part.

For tandem solar cells, the bias illumination and bias voltage used for each subcell
☐ Yes ☒ No
Tandem solar cells are not referred in our manuscript.

5. Calibration

Light source and reference cell or sensor used for the characterization

☒ Yes ☐ No

A 150 W class AAA solar simulator (XES-40S1, SAN-EI) was used as the simulated AM 1.5G standard light source for photovoltaic measurements, and the light intensity of our solar simulator for J-V measurements was calibrated by a standard monocrystalline silicon diode with a KG-5 filter.

Confirmation that the reference cell was calibrated and certified

☒ Yes ☐ No

The light intensity was calibrated by a NIM calibrated standard monocrystalline silicon diode with a KG-5 filter before each measurement.

Calculation of spectral mismatch between the reference cell and the devices under test

☐ Yes ☒ No

No spectral mismatch calculation was performed.

6. Mask/aperture

Size of the mask/aperture used during testing

☒ Yes ☐ No

0.07 cm2

Variation of the measured short-circuit current density with the mask/aperture area

☐ Yes ☒ No

No significant variations were observed.

7. Performance certification

Identity of the independent certification laboratory that confirmed the photovoltaic performance

☐ Yes ☒ No

No certification are conducted in this work since no record efficiency is reported. We focused on investigating the interaction between carriers and defects, along with the phonon scattering process in terms of the defect capture cross-section based on a superior perovskite film quality in the devices instead of reaching the highest certified device performance. And device efficiencies of the perovskite solar cells were credited by an accredited PV Metrology Laboratory of NIM (National Institute of Metrology, China) in our previous work.

A copy of any certificate(s)
Provide in Supplementary Information

☐ Yes ☒ No

No certification are conducted in this work.

8. Statistics

Number of solar cells tested

☒ Yes ☐ No

We tested 100 cells.

Statistical analysis of the device performance

☒ Yes ☐ No

We showed the statistics in Fig. 5e,f, Supplementary Fig. 17 and Supplementary Table 1.

9. Long-term stability analysis

Type of analysis, bias conditions and environmental conditions
For instance: illumination type, temperature, atmosphere humidity, encapsulation method, preconditioning temperature

☒ Yes ☐ No

The test for stability was performed on unencapsulated devices under continuous 1 sun-equivalent illumination provided by the solar simulator in a N2-filled glove box for 120 hours. The cells were sustained at room temperature by cooling with a fan. Every 4 hours, the J-V scan was measured using a Keithley 2400 SourceMeter. J-V curves were then analyzed to extract the relevant parameters.The result was presented in Supplementary Fig. 16.
